# Bacillus velezensis 5113 Induced Metabolic and Molecular Reprogramming during Abiotic Stress Tolerance in Wheat

**DOI:** 10.1038/s41598-019-52567-x

**Published:** 2019-11-08

**Authors:** Islam A. Abd El-Daim, Sarosh Bejai, Johan Meijer

**Affiliations:** 1 Department of Plant Biology, Uppsala Biocenter, Swedish University of Agricultural Sciences and Linnean Center for Plant Biology, SE75007 Uppsala, Sweden; 2 Department of Microbiology, Soils, Water and Environment Research Institute, Agricultural Research Centre, Giza, Egypt; 3 Institute of Biology, Environmental and Rural Sciences (IBERS) Aberystwyth University, Aberystwyth, UK

**Keywords:** Secondary metabolism, Applied microbiology

## Abstract

Abiotic stresses are main limiting factors for agricultural production around the world. Plant growth promoting rhizobacteria (PGPR) have been shown to improve abiotic stress tolerance in several plants. However, the molecular and physiological changes connected with PGPR priming of stress management are poorly understood. The present investigation aimed to explore major metabolic and molecular changes connected with the ability of *Bacillus velezensis* 5113 to mediate abiotic stress tolerance in wheat. Seedlings treated with *Bacillus* were exposed to heat, cold/freezing or drought stress. *Bacillus* improved wheat survival in all stress conditions. SPAD readings showed higher chlorophyll content in 5113-treated stressed seedlings. Metabolite profiling using NMR and ESI-MS provided evidences for metabolic reprograming in 5113-treated seedlings and showed that several common stress metabolites were significantly accumulated in stressed wheat. Two-dimensional gel electrophoresis of wheat leaves resolved more than 300 proteins of which several were differentially expressed between different treatments and that cold stress had a stronger impact on the protein pattern compared to heat and drought. Peptides maps or sequences were used for database searches which identified several homologs. The present study suggests that 5113 treatment provides systemic effects that involve metabolic and regulatory functions supporting both growth and stress management.

## Introduction

Plants experience many abiotic stress conditions during their life cycle that need to be handled in order to survive. Plants responses to abiotic stress are very complex and require modulation in the expression of numerous genes^1,2^. Such genes support stress management either directly, through various factors such as chaperones and osmotic regulators or indirectly, through transcription regulation and signaling^1,2^. In the case of biotic stress there are many specific receptors in cell walls or intracellularly, for example for pathogen-associated molecular patterns and effectors that results in more or less specific responses^3^. Plant recognition of abiotic stress is more intricate and involves various sensing systems for example associated with water status and reactive oxygen species (ROS)^2,4^. Abscisic acid (ABA) is a key player involved in abiotic stress responses and affects the expression of several genes with different functions to mediate systemic stress tolerance^5^. In addition, a number of compounds such as sRNA, peptides and metabolites act as systemic signals to counteract the stress throughout the plant^5,6^.

The responses to abiotic stress include metabolic regulations which often requires wide changes in the concentration, composition, and distribution of both primary and secondary metabolites. Plant primary metabolites, such as amino acids and carbohydrates, are crucial for plant growth and development, while secondary metabolites such as alkaloids and flavonoids are utilized for plant defense^7,8^. Plant responses to abiotic stress factors are costly and during evolution plants have developed an elaborate balance between investments in stress management or growth and reproduction. Various receptors, e.g. for light, direct carbon allocation and transition between different states^9^. However, mild stress can actually improve the management of later more severe stress situations^10^.

Stress tolerance in plants can be improved through treatment of plants or seeds with certain natural and synthetic compounds or microorganisms^11,12^. When this stimulation process of the latent resources of plant defence is only manifested upon stress challenge, it is referred to as priming^12^. Plant growth promoting rhizobacteria (PGPR) are a group of bacteria that can enhance plant growth and productivity^11^. Several PGPR strains can also stimulate abiotic stress tolerance in plants referred to as priming of induced systemic tolerance (IST)^13,14^. Examples of IST include tolerance to salt and drought stress^15,16^, heat stress^17^ and cold stress in wheat^18^. The PGPR based priming phenomenon seems to operate for many plants of agricultural interest, although useful PGPR strains may need to be established for a specific crop variety. Most PGPR are root colonizers but provide a systemic effect for the whole plant^11^. Plant responses to PGPR are complex involving molecular modifications as well as metabolic modulation^1,2,11^. Bacteria based priming of induced systemic resistance to pathogens have identified a number of key components like jasmonic acid, MYC2 and MYB72 needed for priming in plants^19^. However, it is still not fully understood how PGPR prime abiotic stress tolerance. Mechanisms suggested include changes of phytohormone levels through PGPR release of hormones or microbial ACC deaminase activity that decrease ethylene levels^20,21^. More recently, system biology approaches and omics analysis of transcripts, proteins and metabolites have increased our understanding of the molecular responses involved in stressed plants as well as of plant-PGPR interactions^22–24^.

The metabolic and physiological changes caused by PGPR are expected to be driven by molecular tuning that involves changes of protein abundance and post-translational modifications. Hence, combining proteomics and metabolomics profiling for stressed, non-stressed and PGPR-treated plants can help to identify metabolic and molecular modulations involved in beneficial plant microbe interactions under stress conditions and assist in elucidation of the nature of the protection. Proteomic and metabolic studies for drought stress and PGPR responses in crops have been reported for some plants^25,26^. Still, the effect of beneficial microbes on protein expression and metabolite accumulation in PGPR-treated crop plants remains poorly explored especially in plants exposed to temperature stresses.

The PGPR *Bacillus velezensis* UCMB5113 (referred to as 5113) was earlier found to confer improvement of abiotic stress tolerance to wheat^17,27,28^, which formed a basis for more molecular and metabolic analysis of the underlying metabolic and molecular events. We recently employed cDNA-AFLP transcript profiling strategy to identify molecular factors involved in the ability of 5113 to mediate heat, cold and drought stresses tolerance in wheat^29^. We reported that 5113 treatment resulted in both up- and down-regulation in transcripts related to plant growth promotion and stress responsive factors. This study shade more light on metabolic and molecular components related to 5113-mediated abiotic stress tolerance in wheat. The present study is aimed to understand the 5113-mediated programming of metabolic pathways during abiotic stress tolerance in wheat. Further, we also aimed to identify differentially expressed proteins in the leaves of wheat seedlings treated with this beneficial bacterium 5113 and exposed to heat, cold/freeze or drought stresses using protein profiling by two-dimensional gel electrophoresis and MS/MS analysis.

## Results

### Wheat treated with 5113 survived abiotic stress treatments

**Figure 1:**
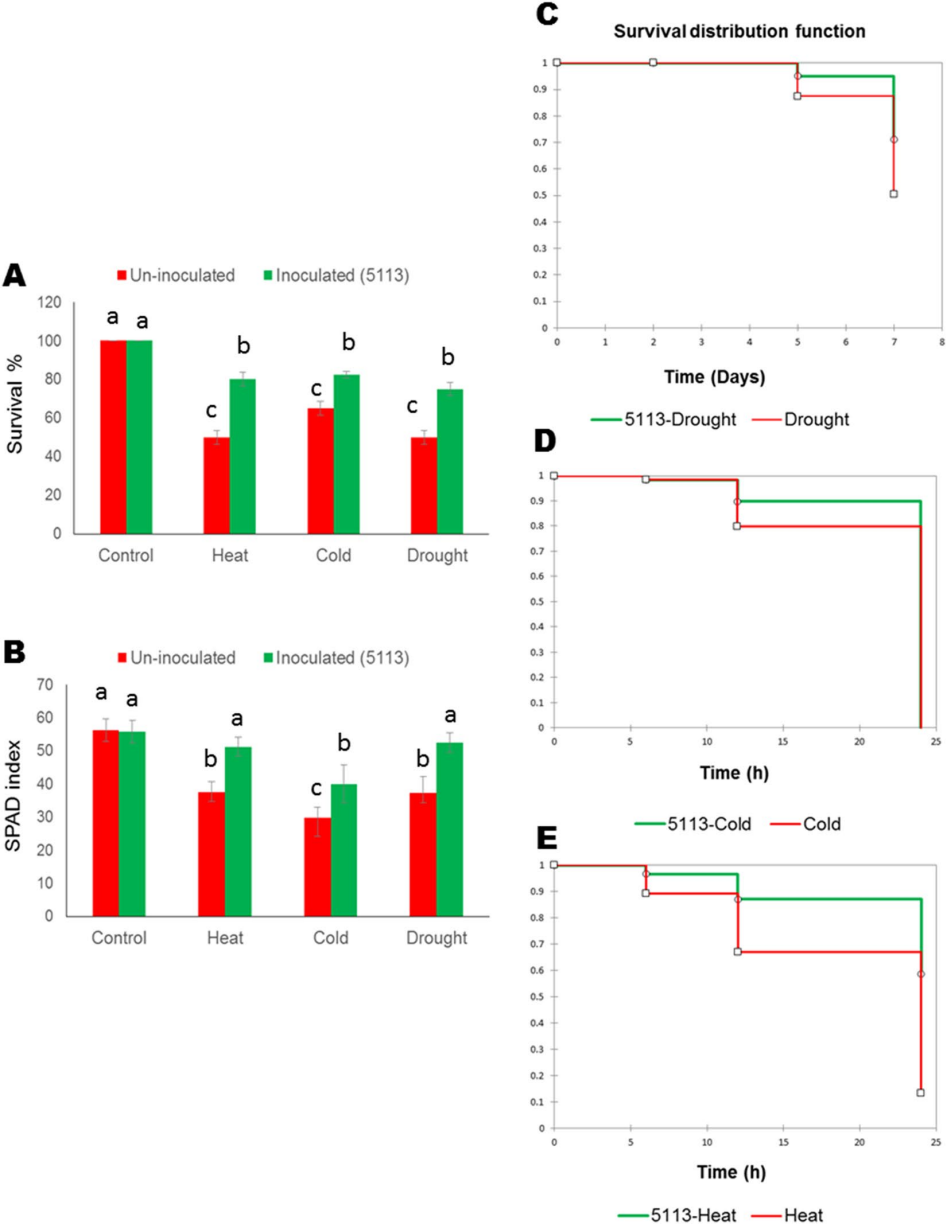
Responses of 5113-treated wheat seedlings to heat stress (12 h 45°C), cold stress (12 h −5°C) and drought stress (7 days without water). (**A**) Survival % (calculated for 2 plant groups (20 plant each), (**B**) SPAD index and Kaplan Meier survival function for 5113-treated drought-stressed (**C**), cold-stressed (**D**) and heat-stressed wheat seedlings (**E**). Bars indicate standard deviation between 3 replicates (5 for SPAD index). Treatments labelled with identical letters are not significant at *p* < *0.05*.

Survival of 5113-treated wheat seedlings was monitored after three days of recovery after exposure for 7 days of drought stress or 12 hours of heat (45 °C) or cold/freezing (−5 °C) stress and compared with their corresponding uninoculated seedlings. More than 70% of 5113-treated wheat seedlings were able to survive stress treatments, which was significantly higher compared to 5113-untreated plants (Fig. 1A). Coupled with significantly improved survival, 5113-treated seedlings also showed significantly higher SPAD index in all stressed wheat plants after three days of recovery (Fig. 1B). Kaplan–Meier survival function analysis suggested that 5113-treated wheat seedlings have significantly higher probability to survive 7 days of drought stress compared to untreated wheat (Fig. 1C). The ability of 5113 to improve wheat seedlings survival after exposure to temperature stress seemed to be higher for heat stress with a significant probability for 5113-treated seedlings to survive 24 hours of heat stress compared to null probability for seedlings exposed to 24 hours of cold stress (Fig. 1D,E).

### Minor effects on the plant ascorbate-glutathione redox cycle

The activities of the four enzymes steering the ascorbate-glutathione pathway were assayed in 5113-stressed wheat leaf tissues and compared to the unstressed controls. Principal component analysis (PCA) was able to discriminate between stressed and unstressed samples, however unstressed non-inoculated samples clustered with unstressed 5113-treated plants suggesting that 5113 had minor impact on the ascorbate-glutathione pathway under normal condition (Fig. S1A). As expected, heat, cold and drought stress treatments caused a significant increase in the activities of ascorbate peroxidase (APX), dehydroascorbate reductase (DHAR), mono-dehydroascorbate reductase (MDHAR), and glutathione reductase (GR) (Fig. S1B–E). Under stress conditions the effect of 5113 treatment on the four enzymes varied and seemed to depend on the stress type. For instance, 5113 significantly reduced APX activity in heat-stressed wheat while a noticeable but not significant reduction was recorded for drought and cold stress (Fig. S1B).

### Profiling of wheat leaf metabolites

**Figure 2:**
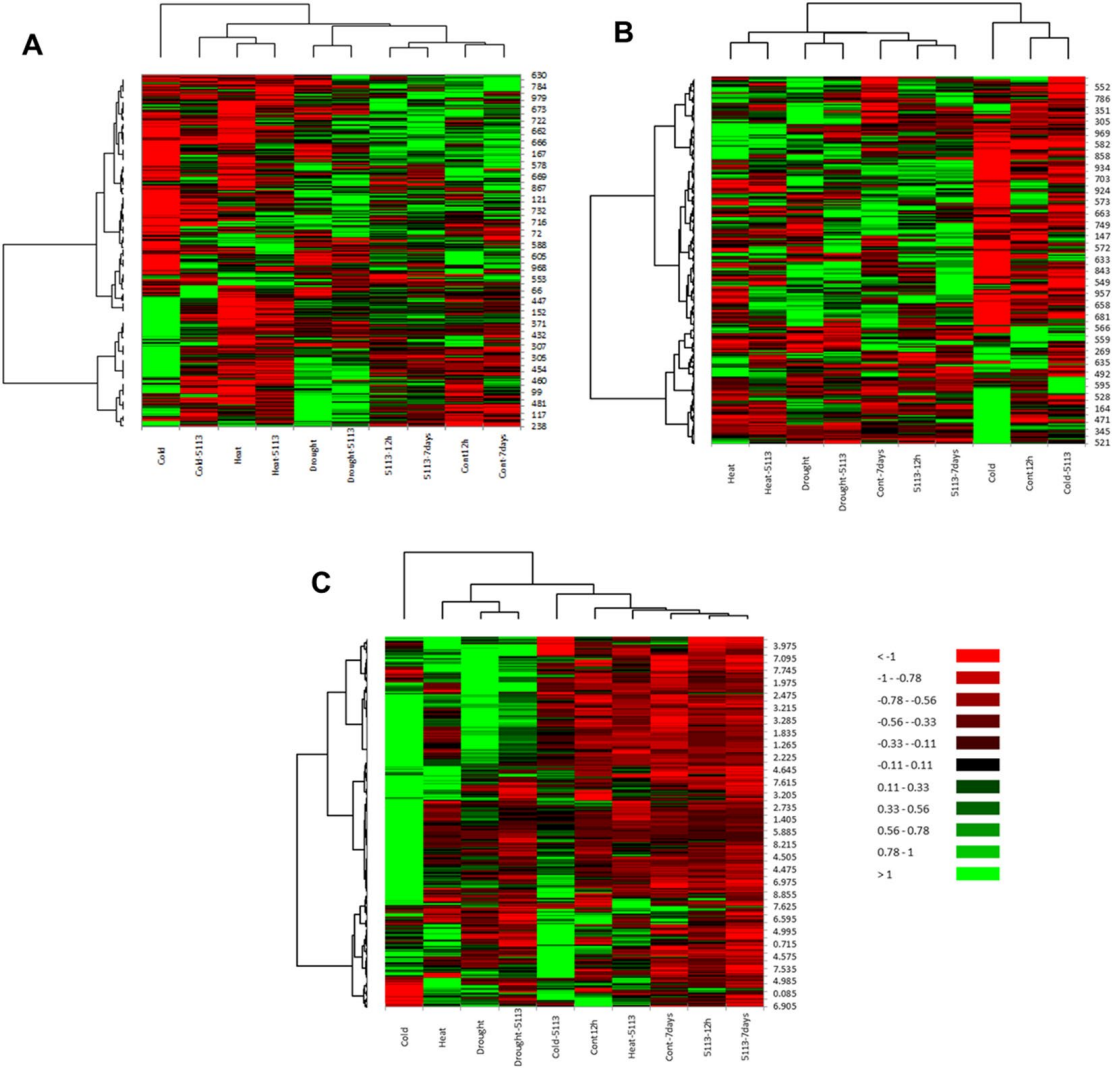
Heat maps for the leaves metabolite profiles of 5113-treated wheat seedlings after exposure to heat stress (12 h 45°C), cold stress (12 h −5°C) and drought stress (7 days without water). Different metabolite profiling approaches were used. Positive mode ESI-MS (**A**), negative mode ESI-MS (**B**) and NMR (**C**). The heat maps represent the average of 9 data points (three biological samples and three technical repeats) and were generated based on Pearson and Ward for distance measure and clustering using XLSTAT package. Numbers right to each heatmap represents potential metabolites.

Metabolite profiling was conducted on leaves of unstressed (controls 12 h and 7 days) as well as stressed (heat (12 h), cold (12 h) and drought (7 days)) 5113-treated and 5113-untreated wheat seedlings using two different profiling approaches (NMR and ESI-MS in positive and negative modes). Data obtained from both metabolite profiling techniques were further analyzed for hierarchical clustering and heat maps were created for visualization of all data sets by XLSTAT (Fig. 2). Data derived from the two different metabolite profiling approaches resulted in relatively different outcomes. Heat maps generated for the metabolite profile obtained using positive mode ESI-MS showed a clear separation between stressed and unstressed samples. Both ESI-MS modes and NMR metabolite profiling showed that stress resulted in drastic changes on wheat leaf metabolites finger prints. The analysis methods resulted in different maps providing complementary sample information. For example MS profiles for cold-stressed samples showed different but mostly negative effects on metabolite levels where 5113 treatment changed the pattern (Fig. 2A,B). On the other hand, NMR metabolite profiling confirmed that cold stress had a pronounced effect on the metabolite profile, but unlike ESI-MS, showed increased metabolite accumulation in wheat leaves while cold-stressed samples treated with 5113 resulted in more down-regulated matabolites. (Fig. 2C).

### Metabolic responses to 5113 treatment in unstressed wheat

**Figure 3:**
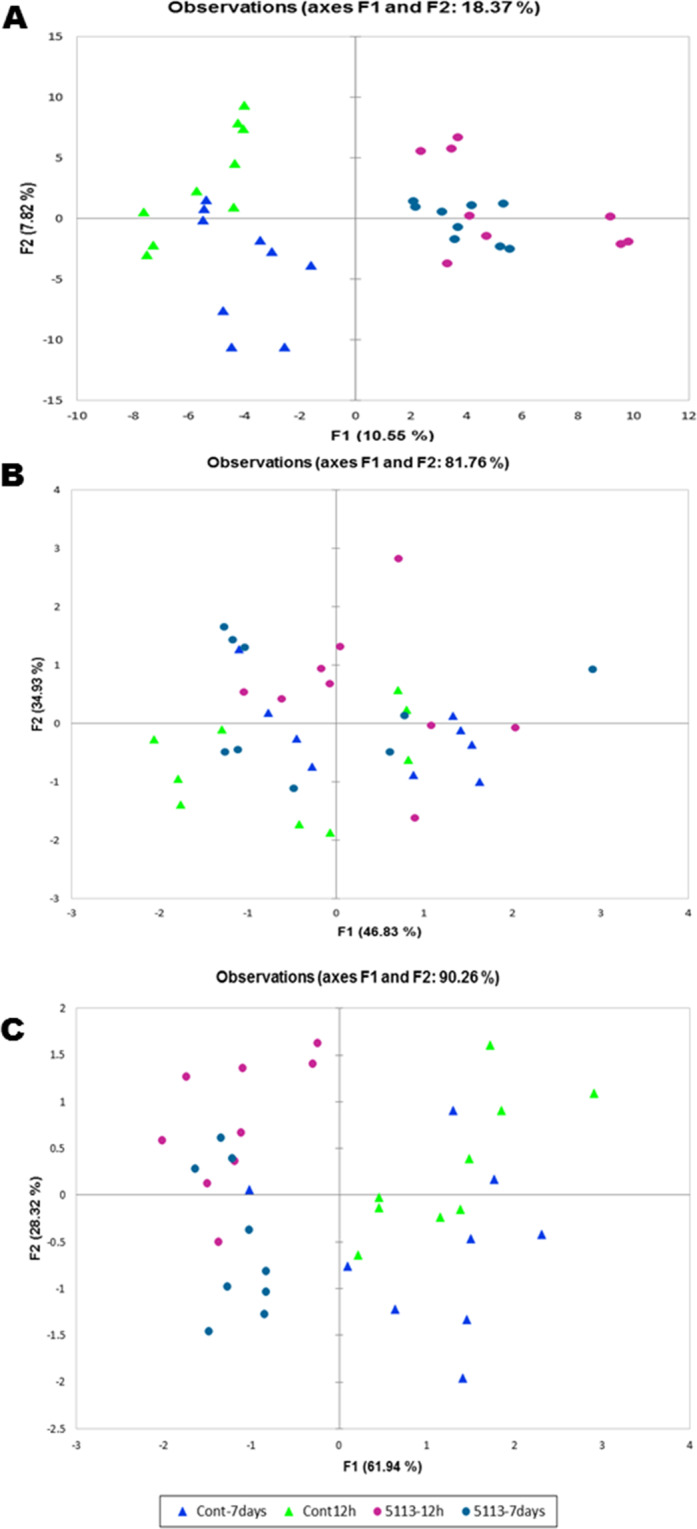
Principle component analysis (PCA) of the metabolite profiles determined in the leaves of unstressed 5113 (12 h and 7 days post 5113 treatment) treated wheat seedlings. Positive mode ESI-MS (**A**), negative mode ESI-MS (**B**) and NMR (**C**). All treatments in these analyses were represented by 9 data points (three biological samples and three technical repeats).

PCA for the metabolite profiles obtained using positive mode ESI-MS was able to separate between 5113-treated and untreated seedlings, but separation was not found between the 5113-treated seedlings at 12 h (served as 5113 control for heat and cold stresses) and the 5113-treated seedlings at 7 days (served as 5113 control for drought stress) (Fig. 3A). Unlike the positive mode ESI-MS, PCA analysis conducted on the metabolic profiles obtained using the negative mode did not discriminate between 5113-treated and untreated seedlings (Fig. 3B). NMR metabolite profiling confirmed the pattern found by the positive mode ESI-MS, which was evident by the ability of PCA to differentiate between 5113-treated and untreated seedlings (Fig. 3C).

**Figure 4:**
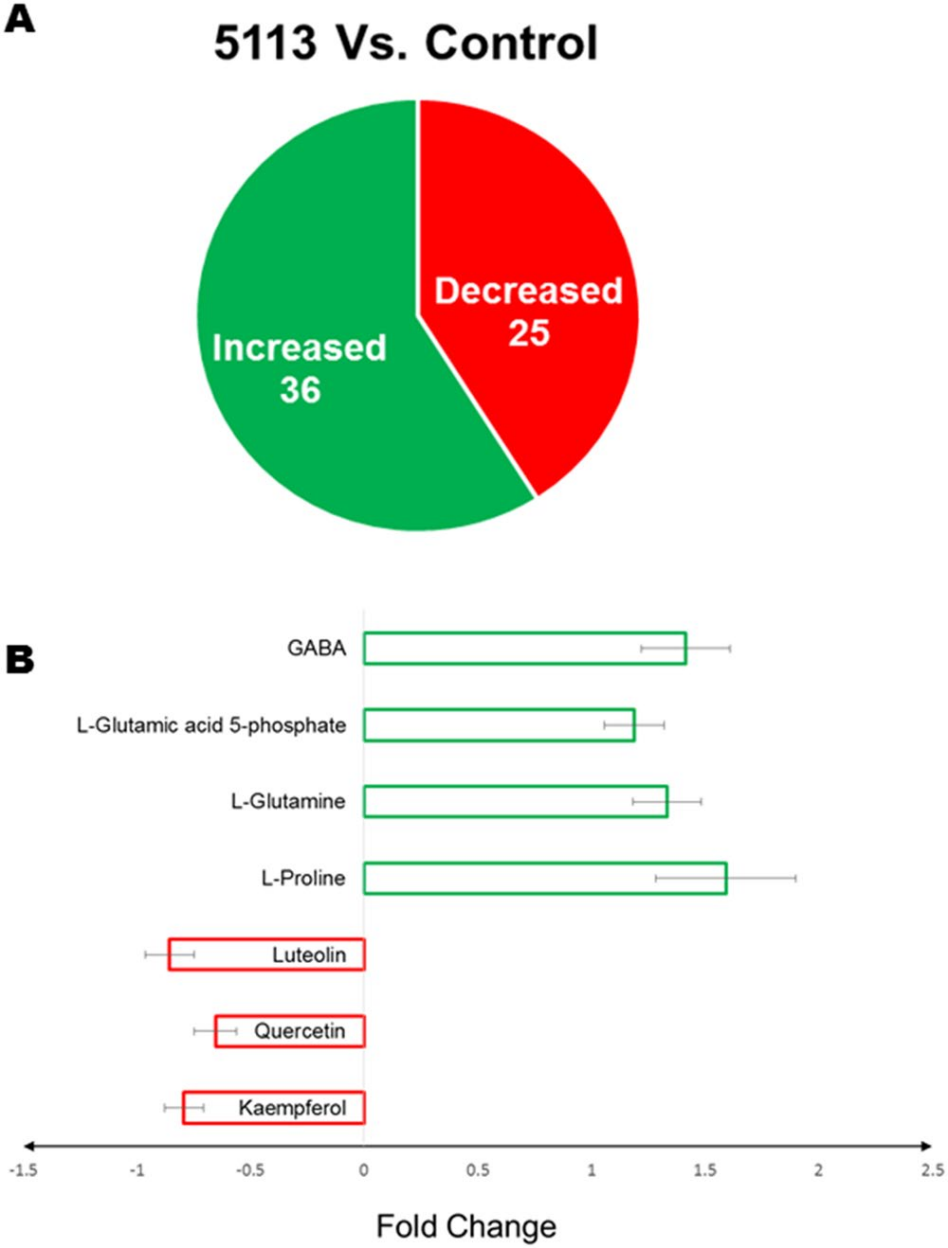
Differential metabolite accumulation in the leaves of 5113-treated unstressed wheat seedlings. (**A**) Metabolites showing significant *(p < 0.05*) increase or decrease accumulation. (**B**) Fold change (relative to control unstressed treatment) in the accumulation of top metabolites showing significant *(p* < *0.05*) differential accumulation. Bars indicate standard deviation between 9 data points (three biological samples and three technical repeats).

Data obtained from the positive mode ESI-MS was used to determine which metabolites showed significant differential accumulation between 5113-treated and untreated wheat seedlings. We found that 5113 treatment caused a significant difference (*p* < *0.05*) in the accumulation of 61 metabolites, 36 showing increased accumulation and 25 showing decreased accumulation (Fig. 4A). Results presented as fold change of some metabolites representing differential accumulation is presented in Fig. 4B. Treatment with 5113 was found to increase the accumulation of several amino acids such as L-proline and L-glutamine as well as γ-aminobutyric acid (GABA) (Fig. 4B). Metabolic pathway analysis suggested that the metabolites showing increasing accumulation in response to 5113 may be involved in several metabolic pathways where alanine, aspartate and glutamate metabolism were most affected by 5113 treatment (Table 1). On the other hand, metabolites showing decreased accumulation in 5113-treated seedlings were also found to be involved in several metabolic pathways. However, the most affected metabolic pathway was the flavonoid biosynthesis pathway (Table 1).

**Table 1:** List of metabolic pathways (in alphabetical order), linked with identified metabolites showing significant (*p* < *0.05*) accumulation in the leaves of wheat seedlings treated or not with *B*. *velezensis* 5113 and exposed to heat (H), cold (C) or drought (D) stress. Controls represent unstressed plants (US).

Metabolic Pathway^a^	Total^b^	Untreated						5113-treated							
		H		C		D		US		H		C		D	
Alanine, aspartate and glutamate metabolism	**21**	−4c	+d1	−3	+1	−1	+3	−1	+4	−1	+1	−1	+1	−1	+3
Amino sugar and nucleotide sugar metabolism	**37**	−3	0	−6	+9	−2	+9	0	+1	−2	0	0	0	−1	0
Aminoacyl-tRNA biosynthesis	**67**	−3	+1	−1	+2	−1	+2	0	+2	−1	+1	0	0	−1	0
Arginine and proline metabolism	**37**	−4	+5	−2	+5	0	+6	−1	+7	−1	+4	−1	+2	0	+3
Ascorbate and aldarate metabolism	**14**	0	0	−2	+1	0	+1	0	+1	0	0	0	+1	0	0
beta-Alanine metabolism	**12**	0	+1	0	+3	0	+1	0	+1	0	0	0	0	0	0
Butanoate metabolism	**20**	−4	+1	−3	+1	−2	+2	0	+1	−2	+1	0	0	−2	0
C5-Branched dibasic acid metabolism	**4**	0	+1	0	+1	0	+1	0	+1	0	+1	0	+1	0	0
Carbon fixation in photosynthetic organisms	**21**	−3	0	−2	+1	−2	+1	0	+1	−1	+1	0	+4	−1	+1
Citrate cycle (TCA cycle)	**20**	−8	0	−4	0	−5	+1	−1	+1	−6	0	−2	+2	−3	+1
Cysteine and methionine metabolism	**35**	−1	+3	−3	+3	−5	+5	0	+1	−2	+1	0	+2	−5	+1
Flavone and flavonol biosynthesis	**8**	−1	+3	0	+3	−1	0	−3	0	−3	+3	−3	0	−3	0
Flavonoid biosynthesis	**37**	−2	+5	−1	+7	−1	+3	−3	0	−5	+3	−3	+2	−3	0
Folate biosynthesis	**16**	0	0	−2	0	−1	0	0	0	0	0	0	0	−1	0
Fructose and mannose metabolism	**18**	−1	0	0	+5	−1	+5	0	0	−1	0	0	0	−1	0
Galactose metabolism	**26**	0	+1	−2	+5	0	+6	0	0	0	0	0	0	0	+1
Glutathione metabolism	**26**	−1	0	0	+1	0	+2	0	0	0	0	0	+1	0	0
Glycerolipid metabolism	**14**	−1	+2	−1	+2	0	+2	0	0	−1	+1	0	+1	0	+1
Glycerophospholipid metabolism	**25**	−4	0	−3	+3	0	0	−1	+1	−4	0	−1	+1	0	0
Glycine, serine and threonine metabolism	**29**	−1	+1	0	+2	−1	+2	0	0	0	+1	0	0	−1	0
Glycolysis or Gluconeogenesis	**25**	−3	0	0	+6	−1	+5	0	0	−3	+1	0	0	−1	0
Glycosylphosphatidylinositol(GPI)-anchor biosynthesis	**10**	0	0	0	+1	0	0	0	+1	0	0	0	0	0	0
Glyoxylate and dicarboxylate metabolism	**17**	−4	+1	−3	0	−3	+2	0	0	−3	+2	−1	0	−3	+1
Histidine metabolism	**16**	−2	+2	0	+4	−1	+1	0	0	−1	+1	−2	+1	−1	+1
Inositol phosphate metabolism	**17**	−3	0	−2	+4	−2	+4	0	0	−2	0	−1	0	−2	0
Isoquinoline alkaloid biosynthesis	**6**	0	0	0	+2	0	+1	0	0	0	0	0	0	0	0
Lysine biosynthesis	**9**	0	+1	0	+1	0	+1	0	0	0	0	0	0	0	0
Metabolism of xenobiotics by cytochrome P450	**25**	0	+1	0	+1	0	+1	0	0	0	+1	0	+1	0	0
Methane metabolism	**11**	0	0	−1	+1	0	+1	0	0	0	+1	0	0	0	0
N-Glycan biosynthesis	**31**	0	0	0	+1	0	0	0	0	0	0	0	0	0	0
Nicotinate and nicotinamide metabolism	**10**	0	+1	−1	+2	−1	+3	0	+1	0	+1	0	0	−1	0
Nitrogen metabolism	**16**	−3	0	−2	0	−1	+1	0	+2	0	+1	0	+1	−1	0
Pantothenate and CoA biosynthesis	**16**	0	+1	0	+2	0	+1	0	+1	0	0	0	0	0	0
Pentose and glucuronate interconversions	**10**	−2	0	−4	+3	−2	+4	0	0	−2	0	0	0	−1	0
Pentose phosphate pathway	**17**	−3	0	−4	+9	−1	+8	0	0	−3	0	0	0	−1	+1
Phenylalanine metabolism	**11**	−2	0	−4	0	−2	+1	0	0	−1	0	0	0	−2	+1
Phenylalanine, tyrosine and tryptophan biosynthesis	**22**	−2	0	−1	+4	0	+3	−1	0	−2	0	−1	0	0	0
Phenylpropanoid biosynthesis	**31**	−2	0	−2	+3	−2	+1	0	+1	−1	0	0	+1	−2	0
Porphyrin and chlorophyll metabolism	**33**	−2	+1	0	0	0	+1	0	+1	0	+1	0	0	0	0
Propanoate metabolism	**14**	−1	0	−1	0	−1	0	0	0	0	0	0	0	−1	0
Purine metabolism	**55**	−13	+3	−9	+11	−6	+12	−2	+4	−7	+1	−9	0	−4	+5
Pyrimidine metabolism	**39**	−12	+6	−5	+7	−3	+5	−2	+6	−7	+4	−5	+1	−2	0
Pyruvate metabolism	**20**	−3	+1	−4	0	−2	+1	−1	0	−3	+1	−1	0	−2	+1
Riboflavin metabolism	**9**	−1	0	−1	+2	0	+1	0	+1	0	+1	0	0	0	0
Selenoamino acid metabolism	**18**	−3	+1	0	+4	0	+2	0	0	−1	0	0	+1	0	0
Sphingolipid metabolism	**13**	−1	0	−1	0	−1	0	−1	0	−1	0	−1	0	−1	0
Starch and sucrose metabolism	**25**	0	0	−3	+4	0	+5	0	0	0	0	0	0	0	0
Stilbenoid, diarylheptanoid and gingerol biosynthesis	**6**	0	0	0	+2	0	+2	0	0	0	0	0	+1	0	0
Sulfur metabolism	**12**	−1	0	−1	0	−2	+2	0	0	−1	0	0	0	−2	+1
Taurine and hypotaurine metabolism	**5**	0	+1	0	+2	0	+1	0	0	0	0	−1	+1	0	0
Terpenoid backbone biosynthesis	**24**	−3	0	−2	+4	−1	+4	0	0	−2	0	−1	+2	−1	0
Thiamine metabolism	**10**	−1	0	−3	+1	−2	0	0	0	−1	0	0	0	−1	0
Tropane, piperidine and pyridine alkaloid biosynthesis	**10**	−1	0	−1	0	−1	0	0	0	0	0	0	0	−1	0
Tryptophan metabolism	**25**	−2	+3	0	+3	0	+2	0	+1	−1	+1	0	+1	−1	0
Tyrosine metabolism	**18**	−1	0	−1	+2	0	+2	0	0	−1	0	0	0	0	+1
Ubiquinone and other terpenoid-quinone biosynthesis	**22**	0	0	0	+2	0	+1	0	0	0	0	0	0	0	0
Valine, leucine and isoleucine biosynthesis	**26**	−4	+3	−2	+2	−1	+3	0	+3	−2	+3	−1	+2	−1	+1
Valine, leucine and isoleucine degradation	**34**	−1	+1	−1	+1	−1	+1	0	+1	−1	+1	0	+1	−1	0
Vitamin B6 metabolism	**11**	−3	0	−4	+3	−1	+3	0	+2	−3	0	0	+1	−1	0
Zeatin biosynthesis	**16**	−1	+1	−2	+1	0	+3	−1	+1	−1	0	−1	+1	0	+2

^a^Pathway name.

^b^Total metabolites involved in that pathways.

^c^Metabolites significantly accumulated in present study (hits).

^d^+, Increased;-, Decreased; 0, no change.

### Differential metabolite accumulation in the leaves of 5113-treated drought-stressed wheat seedlings

Metabolite profiling using ESI-MS (positive and negative mode) and NMR was used to study the variations in the metabolites in 5113-treated wheat after 7 days without water. PCA for the data sets obtained from all profiling approaches clearly separated drought-stressed samples from the unstressed samples. However, no separation was found between 5113-treated and untreated drought-stressed seedlings (Fig. S2).

**Figure 5:**
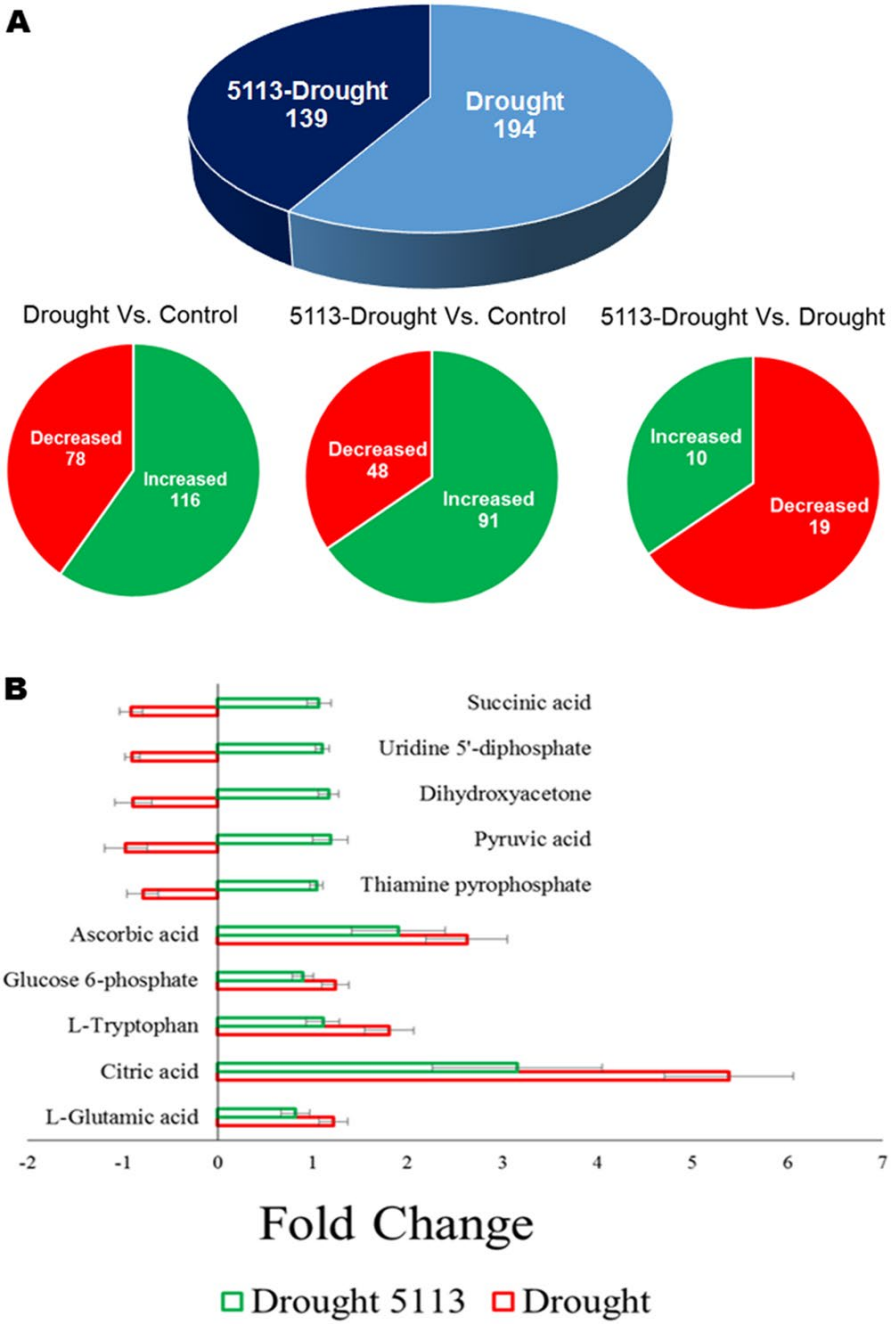
Differential metabolite accumulation in the leaves of 5113-treated drought-stressed wheat seedlings. (**A**) Metabolites showing significant *(p* < *0.05*) increase or decrease accumulation. (**B**) Fold change (relative to control unstressed treatment) in the accumulation of top metabolites showing significant *(p* < *0.05*) differential accumulation. Bars indicate standard deviation between 9 data points (three biological samples and three technical repeats).

A significant (*p* < *0.05*) differential accumulation in the leaves of drought-stressed vs. unstressed wheat seedlings was found for 194 metabolites (Fig. 5A). On the other hand, 139 metabolites showed significant differential accumulation in 5113-treated vs. non-inoculated wheat seedlings under drought stress. We recorded increase in 116 metabolites in drought-stressed seedlings and 91 increased metabolites in 5113-treated drought-stressed seedlings compared to control treatment. Only, 29 metabolites (10 increased and 19 decreased) were found to show significant differential accumulation when 5113-treated drought-stressed seedlings were compared with untreated drought-stressed seedlings.

Metabolite analysis, revealed that drought stress caused great changes in a variety of metabolites including amino acids, organic acids and sugars. Examples representing some metabolites (based on ESI-MS profiles) showing significant differential accumulation between 5113-treated and untreated wheat seedlings under drought stress conditions are shown in Fig. 5B. Metabolic pathway analysis for the metabolites showing significant increased accumulation in drought-stressed wheat seedlings suggested that many of these compounds were involved in metabolism of amino acids (arginine and proline), amino sugars and carbohydrates. Decreased metabolites were found to be involved in several metabolic pathways such as the TCA cycle (Table 1).

### Differential metabolite accumulation in leaves of 5113-treated cold- and heat-stressed wheat seedlings

Cold stress resulted in strong effects on wheat metabolite profiles. In all metabolite profiles (positive and negative modes ESI-MS and NMR) PCA was always able to separate the cold-stressed seedlings from their unstressed counterparts as well as cold-stressed 5113-treated seedlings (Fig. S3). PCA also differentiated cold-stressed 5113-treated seedlings in the metabolite fingerprints obtained by positive and negative modes ESI-MS (Fig. S3A,B). On the other hand, metabolites fingerprinting by NMR was not able to separate cold-stressed 5113-treated seedlings from unstressed seedlings (including 5113-treated) (Fig. S3C).

**Figure 6:**
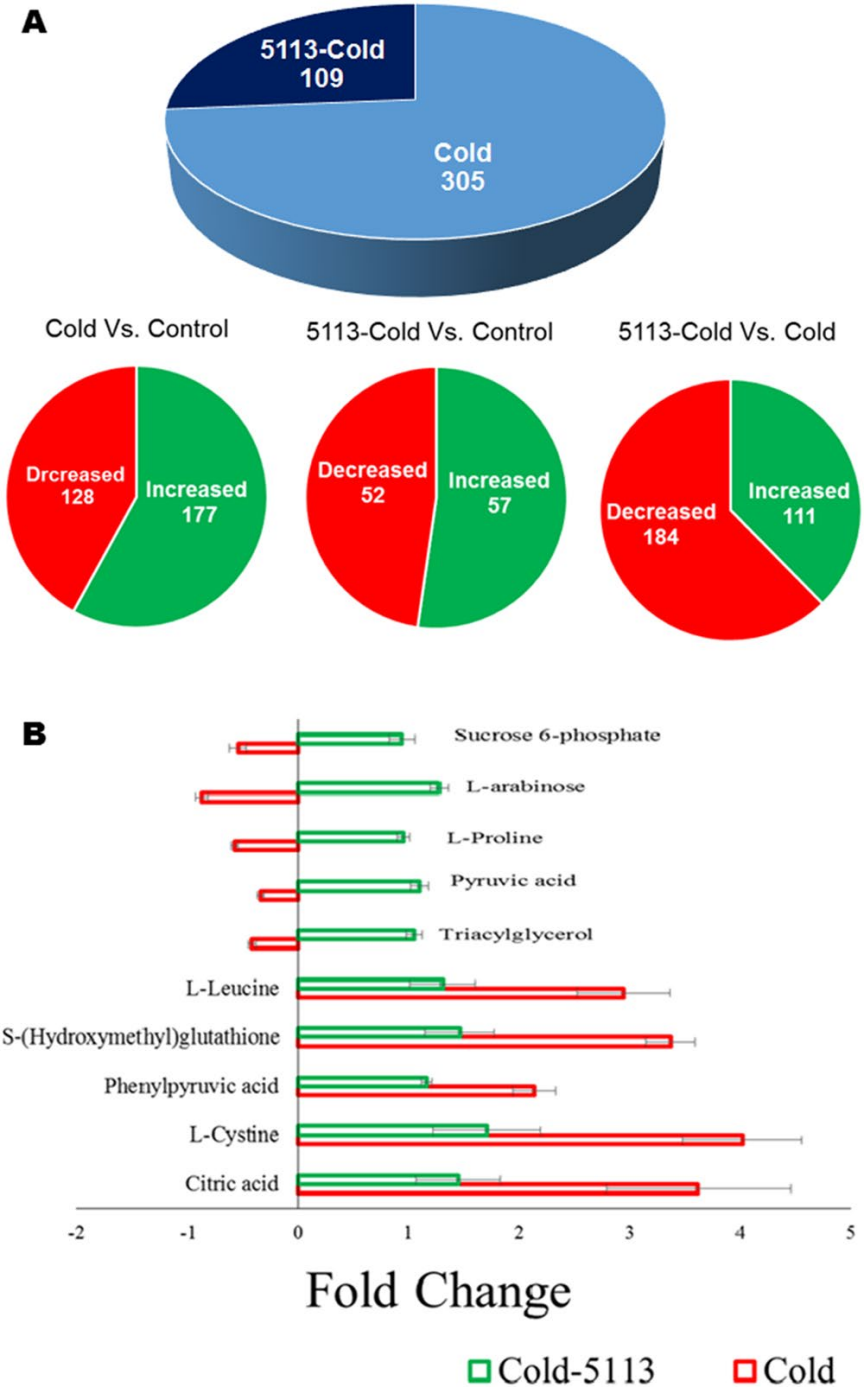
Differential metabolite accumulation in the leaves of 5113-treated and cold-stressed wheat seedlings. (**A**) Metabolites showing significant *(p* < *0.05*) increase or decrease accumulation. (**B**) Fold change (relative to control unstressed treatment) in the accumulation of top metabolites showing significant *(p* < *0.05*) differential accumulation. Bars indicate standard deviation between 9 data points (three biological samples and three technical repeats).

Analysis based on the data obtained from ESI-MS (positive mode), showed significant differential accumulation of 297 metabolites (177 increased and 120 decreased accumulation) in wheat leaves exposed to cold stress. However, metabolites with significant differential accumulation dropped to 109 metabolites (57 increased and 52 decreased accumulation) in the leaves of 5113-treated cold-stressed wheat seedlings (Fig. 6A). Comparing differential accumulation of metabolites between 5113-treated cold-stressed seedlings and 5113-untreated stressed counterparts showed that 5113 treatments resulted in decreasing accumulation of 184 metabolites in the leaves of cold-stressed seedlings (Fig. 6A).

Fold changes of some metabolites representing metabolites with significant (*p < 0.05*) differential accumulation in the leaves of cold-stressed 5113-treated and untreated wheat seedlings are shown in Fig. 6B. The levels of major metabolites including sucrose, proline and pyruvate were decreased in cold-stressed seedlings but found to be increased in 5113-treated cold-stressed seedlings. Other metabolites including citric acid, cysteine and leucine were significantly increasing (up to four-fold) in response to cold stress but showed a lower but significant increase (up to two-fold) in 5113-treated cold-stressed seedlings.

Metabolic pathway analysis for the differentially accumulated metabolites in cold-stressed 5113-treated and untreated seedlings showed impact on a variety of metabolic pathways. The effects of cold stress on the metabolic pathways were grater in the leaves of 5113-untreated seedlings. For instance, several differentially accumulated metabolites in the leaves of cold-stressed seedlings were found to be involved in major metabolic pathways such as amino sugar, nucleotide sugar, fructose and mannose metabolism as well as flavonoid biosynthesis. On the other hand, differentially accumulated metabolites in the leaves of 5113-treated cold-stressed seedlings were not connected with the metabolic pathways linked to cold stress (Table 1).

**Figure 7:**
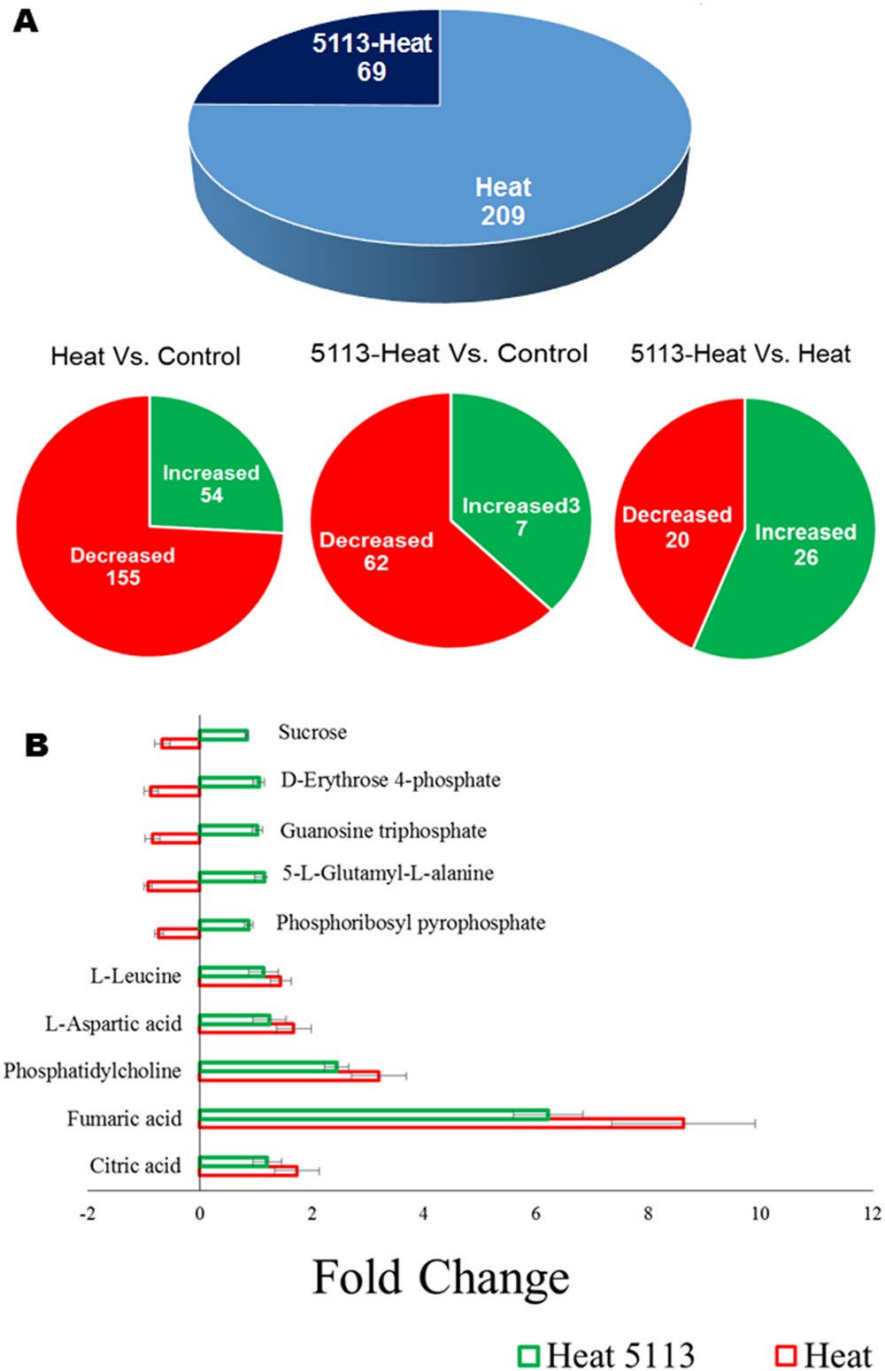
Differential metabolite accumulation in the leaves of 5113-treated and heat-stressed wheat seedlings. (**A**) Metabolites showing significant *(p* < *0.05*) increase or decrease accumulation. (**B**) Fold change (relative to control unstressed treatment) in the accumulation of top metabolites showing significant *(p* < *0.05*) differential accumulation. Bars indicate standard deviation between 9 data points (three biological samples and three technical repeats).

Heat stress resulted in a significant differential accumulation of 209 metabolites (155 decreased and 54 increased) in the leaves of wheat seedlings while 69 metabolites (62 decreased and 7 increased) were found in the leaves of 5113-treated heat-stressed seedlings (Fig. 7A). Further, metabolites with significant differential accumulation (26 increased and 20 decreased) were determined in the leaves of 5113-treated heat-stressed seedlings (compared with 5113-untreated heat-stressed seedlings) (Fig. 7A). Examples of significantly (*p < 0.05*) differentially accumulated metabolites in the leaves of wheat seedlings in response to heat stress and 5113 treatment are presented in Fig. (7B). Metabolic pathway analysis revealed that metabolites with differential accumulation in 5113-treated heat-stressed wheat were involved in several key metabolic pathways including the TCA cycle and carbon fixation as well several amino acid metabolic pathways (Table 1).

### Proteomic analysis

**Figure 8:**
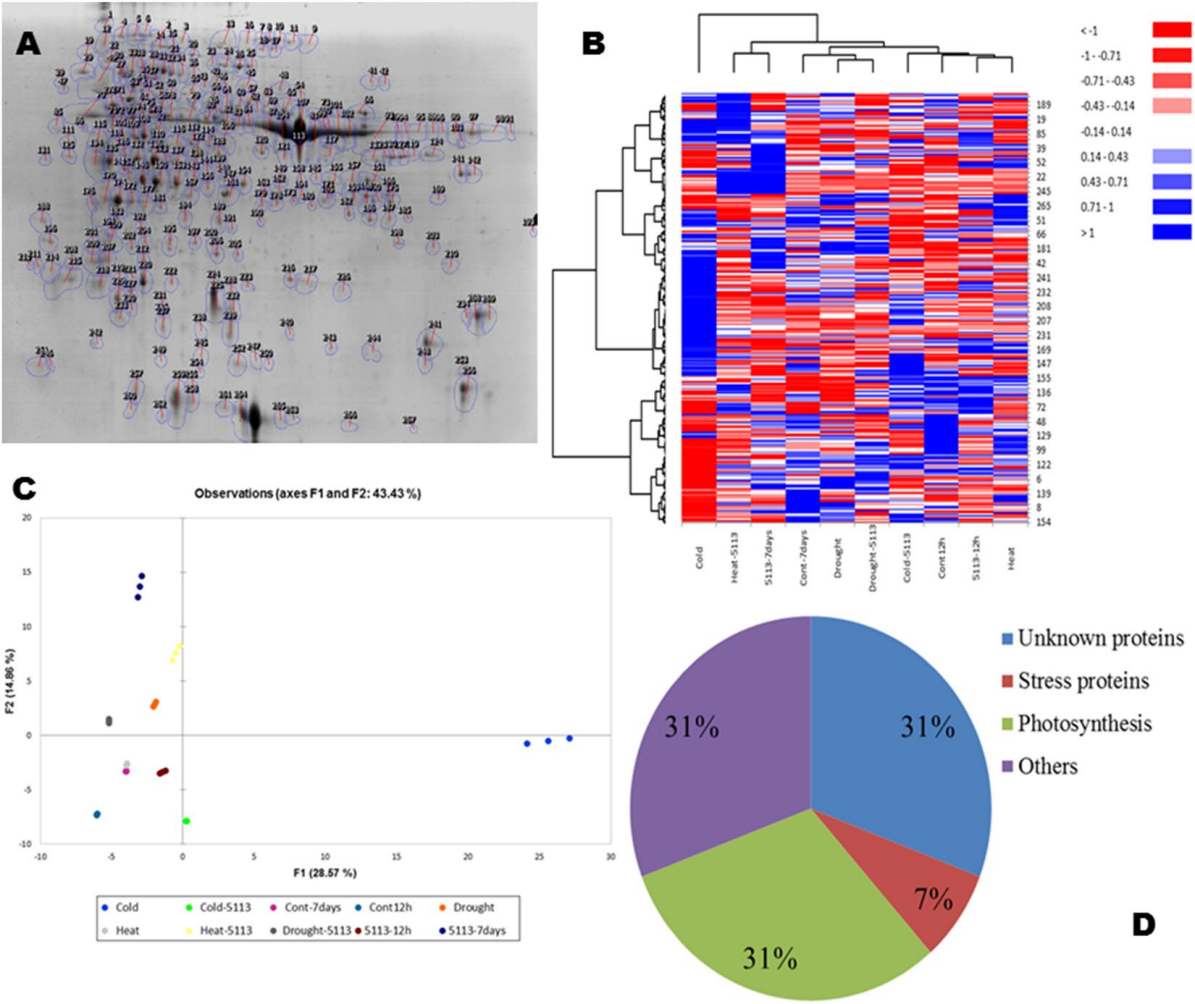
Proteomics analysis and differential regulation of protiens in the leaves of 5113-treated and abiotic stressed (heat, cold and drought stresses) wheat seedlings. (**A**) Two-dimensional gel analyses (reference image representing proteins profiles) generated using image-analysis software Progenesis PG240 (Nonlinear Dynamics, USA). (**B**) Heat maps for the protein profiles of different treatments (numbers right to heatmap represents proteins IDs found on the reference image). (**C**) Principle Component Analysis (PCA) for the protein profiles of different treatments. (**D**) Protein classification based on proteomic analysis Functional distribution of some differentially represented proteins identified after wheat leaf proteomic analysis based on BLAST queries.

Total protein was extracted from leaves of 5113-treated or untreated wheat seedlings grown under heat stress (12 h at 45 °C), cold stress (12 h at −5 °C) or drought stress (7 d without water). Typical two-dimensional gel images (pH 3–10, 15–250 kDa) after Coomassie staining are shown in Fig. S4. Gels were aligned using image analysis and represented by the reference image (Fig. 8A), which showed more than 300 spots. Loading higher amounts of protein caused problems with spot resolution and that big spots like RuBisco masked surrounding spots on the gel. The reference image represents the protein profile of the following treatments; control, 5113-inoculated control, heat-stressed, heat-stressed and 5113-inoculated, cold-stressed, cold-stressed and 5113-inoculated, drought-stressed, and drought-stressed and 5113-inoculated. Profiles for each treatment were processed using Progenesis PG240 which align the gels and assigns, identification number for each spot and determine differential regulation between treatments.

Generally, the effect of 5113 and stress treatments on the peptide distribution on the gels was similar. However, cold stress seemed to be the treatment with the most pronounced effect on the protein pattern (Fig. S4). This was further evident by a clear separation of the cold-stressed samples in PCA and heat map analysis (Fig. 8B,C). The assay was testing basal freezing resistance without adaptation and this treatment seemed to be very tough for the plants reflected in the lack of many typical cold stress associated proteins. MS analysis was used to identify the proteins present in 30 spots showing significant (*p < 0.05*) differential expression among the treatments. Proteins identified based on homology with known proteins in the database (Table 2, Table S1) revealed that many proteins (31%) did not correspond to any known protein. On the other hand, MS analysis showed several homologs to proteins involved in photosynthesis (31%). The remaining identified proteins seemed involved in different functions such as metabolism, stress responses, energy generation and transport (Fig. 8D).

**Table 2:** List of proteins in wheat leaves with significant (*p < 0.05*) different abundance in response to treatment with *B*. *velezensis* 5113 and challenge with heat, cold or drought stress.

ID^a^	NCBI no.	Protein name^b^	kDa	*p*I	E value^c^	Biological Source
155	AAP72270.1	Ribulose-1,5-bisphosphate carboxylase activase	22.5	4.98	1.7e-09	Wheat
238	AAC96315.1	Heat shock protein HSP26	26.9	7.85	3.5e-08	Wheat
194	gi|2443390	Ps16 protein	31.18	4.55	0.017	Wheat
192	gi|4038719	Ribulose-1,5-bisphosphate carboxylase/oxygenase	19.7	8.80	0.00041	Wheat
203	gi|11124572	Triose-phosphate-isomerase	18.99	5.38	1.7e-06	Wheat
202	gi|226533870	cp31BHv	27.04	4.85	0.035	Wheat
206	CAA65042.1	Chlorophyll a/b-binding protein CP26 in PS II	23.17	4.6	0.14	*Brassica juncea*
166	gi|226496743	50 S ribosomal protein L1	37.2	8.69	0.049	*Z. mays*
242	AAB29486.2	Light-harvesting complex I	21.74	5.12	0.035	*H. vulgare*
150	AF080544.1	Amino acid transporter	34.11	5.86	0.33	*Nepenthes alata*
3	gi|2565305	Glycine decarboxylase P subunit	143.5	6.32	6.9e-08	*Tritordeum sp*.
176	gi|148508784	Glyceraldehyde-3-phosphate dehydrogenase	36.8	7.08	0.0055	Wheat
285	gi|134290407	Putative oxygen-evolving complex precursor	21.1	9.72	0.027	Wheat
269	gi|186886530	16.8 kDa Heat-shock protein HSP16.8	16.89	5.82	0.00044	Wheat
260	—	Unknown	19.2	5.9	—	—
240	—	Unknown	22.1	5.4	—	—
218	L12707.1	Photosystem I reaction center subunit II	24.15	9.38	0.015	*H. vulgare*
137	gi|4038719	Ribulose bisphosphate carboxylase activase B	48.0	6.92	4.4e-14	Wheat
179	—	Unknown	32.9	5.92	—	—
55	gi|14017579	ATP synthase beta subunit	55.41	6.41	2.2e-14	*Joinvillea plicata*
219	—	Unknown	24	4.79	—	—
188	—	Unknown	30	8.06	—	—
200	—	Unknown	19.2	5.87	—	—
17	—	Unknown	95	6.7	—	—
25	—	Unknown	82	6.44	—	—
271	gi|022850	Hypothetical protein SORBIDRAFT	19.47	6.45	0.02	*Sorghum bicolor*

^a^Spot number in the reference image.

^b^Homolog of best hit from BLAST search. Unknown samples did not match any sequence in the NCBInr data base.

^c^The E-score from the best hit after BLAST analysis.

Both 5113 and abiotic stress treatments resulted in a differential expression of several proteins in wheat leaves compared to the control (Table 3). The response of proteins with differential expression to 5113 and stress treatments varied a lot (Table 3). Many proteins were upregulated by bacteria and/or stress while other proteins were down-regulated or did not show any response to any treatment.

**Table 3:** Differential abundance of proteins in the leaves of wheat seedlings treated or not with *B*. *velezensis* 5113 and exposed to heat (H), cold (C) or drought (D) stress. Controls represent unstressed plants (US).

ID^a^	Protein name^b^	Untreated						5113-treated							
		H		C		D		US		H		C		D	
155	Ribulose-1,5-bisphosphate carboxylase activase	0 c	0 d	—	3.67	—	3.47	—	2.60	—	2.65	0	0	+	2.71
238	Heat shock protein HSP26	+	2.99	0	0	0	0	+	2.15	0	0	0	0	0	0
194	Ps16 protein	—	2.88	—	2.75	—	2.83	+	2.18	+	2.39	+	2.26	+	2.42
192	Ribulose-1,5-bisphosphate carboxylase/oxygenase	—	2.64	—	3.15	—	2.54	+	2.09	+	2.76	+	2.54	+	2.63
203	Triose-phosphate-isomerase	—	2.87	—	3.01	0	0	+	2.30	+	2.95	+	2.87	+	2.93
202	cp31BHv	+	2.79	—	2.81	—	2.73	+	2.22	+	2.38	+	2.34	—	2.11
206	Chlorophyll a/b-binding protein CP26 in PS II	+	2.31	+	2.67	+	2.11	+	2.39	—	2.12	+	2.51	+	2.43
166	50 S ribosomal protein L1	+	2.09	—	2.49	—	2.61	+	2.23	+	2.54	+	2.32	—	2.06
242	Light-harvesting complex I	+	2.51	+	2.39	+	2.76	+	2.18	+	2.61	+	2.62	+	2.53
150	Amino acid transporter	0	0	—	2.05	—	2.21	+	2.71	—	2.87	+	2.29	+	2.35
3	Glycine decarboxylase P subunit	0	0	—	2.61	—	2.54	—	2.01	0	0	0	0	0	0
176	Glyceraldehyde-3-phosphate dehydrogenase	—	2.91	—	2.86	—	2.37	+	2.80	+	2.97	+	2.75	+	2.85
285	Putative oxygen-evolving complex precursor	—	2.71	—	2.84	—	2.36	—	2.22	—	2.41	—	2.52	—	2.51
269	16.8 kDa Heat-shock protein HSP16.8	+	2.94	0	0	0	0	0	0	+	2.87	0	0	0	0
260	Unknowne	0	0	0	0	0	0	+	2.01	+	2.53	+	2.48	+	2.63
240	Unknown	0	0	0	0	0	0	+	2.39	0	0	0	0	0	0
218	Photosystem I reaction center subunit II	0	0	+	2.51	+	2.69	+	2.61	0	0	0	0	0	0
137	Ribulose bisphosphate carboxylase activase B	0	0	0		0	0	0	0	+	2.11	0	0	0	0
179	Unknown	+	2.84	—	2.31	0	0	0	0	+	2.41	—	2.12	0	0
55	ATP synthase beta subunit	0	0	0	0	0	0	+	2.19	—	2.10	—	2.06	—	2.14
219	Unknown	+	2.61	+	2.11	+	2.34	+	2.00	+	2.31	+	2.39	+	2.34
188	Unknown	+	2.20	+	2.18	+	2.33	+	2.86	+	2.51	+	2.10	+	2.32
200	Unknown	—	2.13	—	2.54	0	0	0	0	+	2.38	+	2.53	+	2.69
17	Unknown	0	0	—	2.35	0	0	0	0	+	2.29	0	0	0	0
25	Unknown	+	2.61	+	2.11	+	2.51	+	2.39	+	2.76	—	2.47	+	2.55
271	Hypothetical protein	—	2.13	—	2.19	—	2.46	—	2.37	—	2.34	—	2.23	—	2.65

^a^Refers to spot numbers on the reference gel.

^b^Homolog of best plant hit from BLAST search. Unknown samples did not match any sequence in the NCBInr data base.

^c^+, Increased; −, Decreased; 0, no change.

^d^Fold changes relative to control unstressed treatments (significant *p < 0.05*).

The effect of both 5113 and abiotic stress treatment were pronounced. For instance, 5113 treatments resulted in an increase in the fraction representing proteins matching already known proteins, corresponding to 64% of the total compared to 16% being increased and 20% unaffected. The majority of the increased proteins in response to 5113 treatments were proteins involved in photosynthesis and metabolism. Several unknown proteins were also found to be upregulated in wheat leaves in response to 5113. On the other hand, 40% of the identified proteins showed increased levels in leaves after exposing wheat seedlings to 12 h of heat stress. Results also showed that 52% of the proteins were down-regulated in leaves under the same conditions. *B. velezensis* 5113 resulted in upregulation of 64% of the proteins in the leaves of heat-stressed wheat. Most of the down-regulated proteins in the leaves of the heat-stressed seedlings were proteins related to photosynthesis. However, it was observed that many of these proteins were not down-regulated in leaves of the 5113-treated seedlings (Table 3). Exposing wheat seedlings to 12 h of cold stress also resulted in down-regulation of proteins related to photosynthesis and other biological functions. However, the majority of the down-regulated proteins were found to be upregulated in the leaves of their 5113 counterparts (Table 3). Moreover, we detected down-regulation of the majority of the same proteins in the leaves of drought-stressed wheat seedlings while the expression was recovered in 5113-treated and drought-stressed seedlings (Table 3).

## Discussion

PGPR have been successfully used to improve abiotic stress tolerance in many plants including wheat^13,14^. Previously, we showed that *B. velezensis* UCMB5113 was able to improve wheat tolerance against heat, cold and drought stress conditions^17,27,28^. In the present study, our finding confirmed our previous reports by providing statistical evident for the ability of 5113 to improve wheat survival under heat, cold and drought stress conditions. Further, the higher SPAD index found in all 5113-treated seedlings suggest that 5113 could potentially preserve the plant photosynthetic machinery by maintaining higher contents of photosynthetic pigments although analysis of photosystem activity is needed to confirm this.

Metabolic regulations are key actions assuring plant survival under abiotic stress conditions^8^. In the present study we used NMR and ESI-MS (positive and negative modes) to determine the metabolite fingerprints in the leaves of 5113-treated and untreated wheat seedlings exposed to heat, cold and drought stresses. Wheat treatment with 5113 resulted in significant effects on the metabolite profiles recorded in the leaves of unstressed wheat seedlings. Moreover, a significant increase in the accumulation of GABA, glutamine and proline was also detected in 5113-treated plants. The non-protein amino acid GABA is known to rapidly accumulate in plant tissues in response to both biotic and abiotic factors^30^. GABA is linked with the maintenance of the carbon-nitrogen balance, the metabolism of amino acids and carbohydrates, and the regulation of plant growth where regulation of anionic transporters seems to be a key event^30,31^. Further, GABA could act as an effective osmolyte without toxic effects and function as ROS scavenging in plant exposed to abiotic stress^32^. It has been reported that GABA is potentially able to prime plant defense/tolerance against biotic and abiotic stress tolerance^33^. For instance, it was reported that GABA priming induced osmotic stress tolerance in *Piper nigrum*^34^. Further, Yong *et al*. reported that exogenous application of GABA was able to induce drought stress tolerance in white Clover and attributed this ability to the association with GABA-shunt, polyamines, and proline metabolism^35^. Hence, in our study, the ability of 5113 to modulate GABA levels in unstressed wheat leaves might suggest that 5113 is able to mediate abiotic stress tolerance through priming in a manner similar to that achieved by exogenous application of GABA. Jakab *et al*., suggested that BABA and GABA priming is based on enhanced ABA accumulation and in turn dependent on ABA signaling^36^. In this connection, we have reported that 5113 priming also might employ ABA signaling based on functional analysis using viral induced gene silencing to mediate abiotic stress tolerance in wheat^29^.

Flavonoids are secondary metabolites synthesized by plants and known to play roles in plant defense and plant-microbe interactions^37^. In the present study we showed that 5113 treatment resulted in decreased accumulation of some metabolites related to flavone and flavonol biosynthesis as well as flavonoid biosynthesis in the leaves of both stressed and unstressed wheat seedlings suggesting a possible 5113 related inhibitory effect on flavonoid biosynthesis in plants. Several studies reported the ability of PGPR to modulate flavonoids in plants^38^. For instance, Chamam *et al*. conducted a metabolic profiling for two rice genotypes treated with two different PGPR *Azospirillum* strains and reported both increased and decreased accumulation of several classes of secondary metabolites with phenolic compounds such as flavonoids and hydroxycinnamic derivatives being the main metabolites affected^39^. They further observed that the ability of *Azospirillum* to modulate plant secondary metabolites were related to the combination of plant genotypes and bacterial strains^39^. Other groups of metabolites significantly affected by stress and 5113 were purines and pyrimidines. These compounds fulfill a number of important functions producing building blocks for nucleic acids, energy intermediates, cytokinins, secondary metabolites and other compounds. While stress treatments showed extensive effects on the levels of various purines and pyrimidines, treatment with 5113 attenuated these effects. Given the central role of purines and pyrimidines and the multiple metabolic pathways possible these changes are not surprising. Another effect that may show up is that salvage pathways become activated that use less energy and also often result in lower pool sizes of metabolites^40^. Further, many plant associated bacteria also contribute to purine and pyrimidine metabolism that may affect plant metabolite levels^41^. PGPR support plants but obviously also profit from plants and can thus also affect metabolism and levels of various metabolites.

There is a well-known tight relationship between amino acids metabolism and abiotic stress responses in plants. For instance, amino acid accumulation has been widely suggested to be directly involved as a protective mechanism in many stressed plants^42^. In the present study, we found evidences for the modulation of metabolic pathways for several amino acids, in the leaves of wheat seedlings exposed to heat, cold and drought stress conditions. Further, amino acid metabolic modifications were detected in the leaves of 5113-treated seedlings whether stressed or not. Our results showed that pathways related to alanine, aspartate, glutamate arginine and proline metabolism were affected by 5113 and stress treatments. For instance, the enrichment analysis used to identify the most relevant metabolic pathways for the metabolites showing significant differential accumulated metabolites suggested that 7 metabolites with increased accumulation in the leaves of unstressed 5113-treated seedlings were connected to arginine and proline metabolism. Several metabolites related to the same pathway were also found to accumulate in all stressed samples. Increased levels of proline due to 5113 treatment could support plant stress tolerance in several ways serving as osmolyte, antioxidant and metal chelator^43^. In this connection, Khan *et al*., reported the ability of three PGPR isolates identified as *B. subtilis*, *B. thuringiensis*, and *B. megaterium* to induce drought stress tolerance in Chickpea. Moreover, by conducting a comparative physiological and metabolic analysis they connected the PGPR ability to mediate drought stress tolerance with complex plant physiological and metabolic modifications including changes in biosynthesis and metabolic pathways of amino acids such as proline, phenylalanine, tyrosine, and tryptophan; alanine, aspartate, and glutamate^26^.

Previous reports have shown that exposing plant to PGPR treatment and abiotic stress factors can result in differential regulation in several genes and proteins which is essential to mount required plant physiological and developmental responses under stress conditions^44–46^. The present study showed that 5113 and stress treatments resulted in both decreased and increased abundance of several proteins connected to several metabolic and stress events and also proteins with unknown function. No sequenced proteins in the wheat leaf samples were derived from the *Bacillus* strain for which the genome sequence and predicted proteome is available^47^. This was expected since this rhizobacterium is a root colonizing strain and analysis by qPCR on oilseed rape tissues confirmed the localization of 5113 to root tissues^48^. In the present study proteomics profiling of wheat leaves sampled from wheat treated with 5113 revealed that the levels of several proteins involved in photosynthesis (ID 194, 192, and 218) were increased. Interestingly, these proteins were found to be down regulated under all tested abiotic stress conditions in non-primed plants suggesting that 5113 treatments could protect the photosynthesis process in stressed plants to support energy and organic compound. Otherwise the photosynthetic apparatus is often compromised under abiotic stress situations^49^. Further, in the present study we also reported significant higher SPAD index readings in 5113-treated wheat seedlings which also could suggest that 5113 was able to protect plant photosynthetic machinery under stress conditions. The ability of the PGPR *P. fluorescens* to protect photosynthesis in drought-stressed rice plant has been reported^50^. The protective ability of this PGPR was suggested to be connected to increased levels of chaperones that support assembly of RuBisCO, which increased the photosynthetic efficiency under drought stress conditions^50^. The ability of PGPR to improve photosynthesis is expected to support both growth promotion and stress tolerance.

The stress induced change of intracellular environments creates great challenges for protein function. Chaperones are essential for folding, assembly and maintenance of many proteins in a functional conformation as well as to prevent aggregation of proteins during stress caused by unfavorable folding conditions. Chaperones as heat shock proteins (HSPs) are crucial for maintaining proteins functional in many cellular processes and compartments^51,52^. The small heat shock protein HSP26 was expressed at higher levels in the leaves of *Bacillus*-treated wheat seedlings, which might indicate a possible pre-stimulation for abiotic stress responses in leaves. However, upon stress challenge the relative abundance was lower suggesting cellular priorities for other proteins. In this connection, we showed by RT-PCR that transcript levels of another small heat shock protein, HSP 17.8, was up-regulated in wheat leaves after treatment with 5113^17^. Using cDNA-AFLP analysis of wheat we also found a heat shock protein to be up-regulated by heat, cold or drought^29^. Several investigations suggested that over-expression of HSP play an important role in acquired stress tolerance in plants^52,53^. Another protein that was more abundant in primed control plants but less abundant in stressed plants, was the ATP synthase beta subunit. ATP synthase subunits have been shown to have differential responses to abiotic stress with no clear pattern^54^.

The different stresses did not provide the same response on the proteins analysed. Heat stress of primed plants resulted in lower levels of an amino acid transporter and a chlorophyll a/b-binding protein compared to drought and cold stress. Drought-stressed and primed plants showed lower abundance of the chloroplastic RNA-binding protein cp31BHv and 50 S ribosomal protein relative to heat and cold stress suggesting compromised chloroplast function. The pattern of differential responses among stressed and primed plants were not the same for non-primed stressed plants showing that priming do somehow change the regulation of protein levels differently as displayed during different kinds of stress. Several sensing systems and transcription factors have been indicated to play major roles during stress adaptation^2,55^ and a few has also been associated with priming events^12^. In a study of wheat stress responses studied by cDNA-AFLP analysis a differential response pattern for different kinds of stress was also observed^29^. This analysis used a limited set of oligonucleotide primers for amplification and only a subset of the products generated was sequenced and transcript levels may not reflect functional protein levels fully. Accordingly these two studies cannot be compared with regards to specific genes although some overall changes were in common concerning e.g. heat shock proteins and photosynthesis.

Treatment with 5113 caused upregulation of triose-phosphate isomerase, glyceraldehyde-3-phosphate dehydrogenase in both control and stressed plants. Improved sugar metabolism contributes to both growth promotion and stress management. Abiotic stress in general is often connected to changes in energy metabolism including carbohydrate metabolism and photosynthesis^54^. Also proteins with unknown function were stimulated by 5113 that would be interesting to identify to better understand the mechanisms of protection. Other proteomic analyses of wheat exposed to abiotic stress have identified various proteins to be affected while effects of PGPR treatment have received less attention. An investigation of transcriptome changes during IST to drought in *Arabidopsis thaliana* primed by *Pseudomonas chlororaphis* O6 found changes in several hormone response pathways due to root colonization but different responses to drought^56^. The *Pseudomonas* strain also increased transcripts for genes related to defence and reactive oxygen species under normal conditions. However, drought responsive signaling genes were actually down-regulated during drought suggesting that the primed protection involves other mechanisms that improve stress tolerance. Kandasamy *et al*., conducted a proteomics analysis of drought-stressed rice after priming with the PGPR *P. fluorescens* and concluded that the protective effects of the bacterium were associated with molecular modulation in multiple processes and mechanisms involving various stress inducible proteins^50^. Moreover, Timmusk *et al*., showed that PGPR inoculation resulted in a significant increase in net assimilation and stomatal conductance in drought-stressed wheat, which was positively correlated with the ability of wheat to survive under drought stress conditions^57^. In this case volatiles released from the PGPR were suggested to play an important role for the effects on the host plant. Further work is apparently needed to identify how PGPR stimulates abiotic stress tolerance in plants and this may result in a set of abiotic stress biomarkers that could facilitate both plant breeding and screening of different PGPR to predict efficacy of IST for different crops^58^. Eventually bacteria with characterized action spectra and mechanisms of action that support abiotic stress tolerance can be explored in crop production similar to products available for growth promotion and disease control supporting more durable food production^59^.

In conclusion, *B. velezensis* 5113 inoculation resulted in a significant metabolic modulation and affected abundance of several proteins in wheat leaves. Our results indicated that 5113 utilized similar metabolic and molecular regulatory strategies to enhance tolerance of wheat exposed to different abiotic stress factors (heat, cold and drought). The success of 5113 to improve abiotic stress tolerance seems to be related to its ability to; 1, Tune the expression of several proteins related to stress defense as well as energy supply. 2, Modulate several metabolic pathways of amino acids. 3, Increase the accumulation of GABA in the leaves of 5113-treated seedlings (before exposure to stress). On the other hand, the ability to promote plant growth could be connected with increased photosynthesis both in the absence and presence of stress conditions. Our results also points to other unknown mechanisms waiting to be uncovered with the advances of gene function analysis technologies.

## Methods

### Plant growth, bacterial inoculation and abiotic stress treatment

Wheat (*Triticum aestivum* L.) cv. olivin was obtained from Scandinavian Seed AB (Lidköping, Sweden). This winter variety was developed for improved winter hardiness and protein content. Grains were surface disinfected as described^60^.

*B. velezensis* subsp. *plantarum* UCMB5113 (earlier referred to as *Bacillus amyloliquefaciens*)^61^ was grown in LB medium for 48 h at 28 °C and after centrifugation and washing the concentration was determined using colony forming unit counts. Wheat grains were soaked in 5113 solution (10^7^ mL^−1^) for 2 h at 28 °C with shaking. Control grains were soaked in water. Grains were sown in sterile soil (Weibulls, Hammenhög, Sweden) in controlled environment with 22/16 °C (day/night), 16/8 h photoperiods with light intensity of 450 µmol m^−2^ s^−1^ and 80% humidity. Seedlings were watered every other day and left to grow for 12 days.

For drought stress, twelve days old wheat seedlings were kept at 22/16 °C (day/night), 16/8 h photoperiods with light intensity of 450 µmol m^−2^ s^−1^ and 20% humidity and allowed to grow for 5 or 7 d without water. Control seedlings were watered every other day. Heat and cold stress used twelve days old wheat seedlings exposed to either 45 °C or −5 °C for 6 and 24 h with 16/8 h photoperiods with light intensity of 450 µmol m^−2^ s^−1^ and 80% humidity. Control seedlings were allowed to grow under normal conditions.

### Plant survival

At each sampling period (0, 6, 12 and 24 hours for heat and cold stresses and 0, 2, and 7 days for drought stress), 40 seedlings were randomly selected, randomly divided into two groups (20 plants/group) and kept at normal conditions to allow recovery. After 3 days, the recovered seedlings (with at least 1 erect green leaf) were counted as survived and subjected to survival analysis using the Kaplan–Meier survival function^62^ as well as survival %^27^.

### Leaf chlorophyll content

SPAD chlorophyll meter (Spad-502 Konica Minolta, Japan) was used for the determination of chlorophyll content in plant leaves of 5113-treated (12 hours heat or cold stressed) and 7 days drought-stressed as well as stressed and unstressed controls wheat seedlings. SPAD index was recorded for 5 seedlings (5 times/each seedling)^63^.

### Antioxidant enzyme assays

The enzymes ascorbate peroxidase (APX), mono-dehydroascorbate reductase (MDHAR), dehydroascorbate reductase (DHAR) and glutathione reductase (GR) involved in the plant ascorbate-glutathione cycle were assayed using the microplate reader method as described by Murshed *et al*.^64^. Frozen plant material (200 mg) sampled from 5113-treated (12 hours heat or cold stressed) and 7 days drought-stressed seedlings as well as stressed and unstressed controls wheat seedlings were homogenized in 1 mL extraction buffer (50 mM MES/KOH buffer (pH 6.0) containing 40 mM KCl, 2 mM CaCl_2_ and 1 mM L-ascorbic acid). The homogenate was centrifuged at 14,000 g for 10 min at 4°C and the supernatant was analyzed for enzyme activities using a FLUOstar Omega, microplate reader (BMG LABTECH; Germany).

### Metabolomics analysis

Leaves from 5113-treated and stressed (12 h heat and cold) wheat seedlings were subjected to metabolomics analysis conducted at the National Centre for Plant and Microbial Metabolomics in Rothamstead, UK. According to the methods described by Ward *et al*.^65,66^. Freeze-dried wheat leaves (15 mg/sample) were weighted into 2 mL Eppendorf tube. D2O:CD3OD (1 mL, 80:20) containing 0.05% w/v TSP-d4 (sodium salt of trimethylsilylpropionic acid) was added to each sample. The contents of the tube were mixed thoroughly and then heated at 50 °C in a water bath for 10 min. After cooling, the samples were spun down in a micro-centrifuge for 5 min. Of the supernatant 750 μL were added to a 5 mm NMR tube for ^1^H NMR, while another 50 μL aliquot was mixed with 950 μL 80:20 D2O:CD3OD for parallel ESI-MS analysis.

All ^1^H-NMR spectra were acquired under automation at a temperature of 300 K on a Bruker Avance spectrometer operating at 600 MHz ^1^H observation frequency using the SEI 5 mm probe. The WATERSUP pulse sequence was used with a relaxation delay of 5 seconds. Each spectrum consisted of 128 scans of 32,000 data points. The spectra were automatically Fourier transformed using an exponential window with a line broadening value of 0.5 Hz, phased and baseline corrected within the automation software. ^1^H-NMR chemical shifts in the spectra were referenced to d4-TSP at δ 0.00. ESI-MS data was collected on a Bruker Esquire 3000 mass spectrometer. The ^1^H-NMR spectra were automatically reduced to ASCII files using AMIX (Analysis of MIXtures software v.3.0, Bruker Biospin). Spectra were scaled to d4 TSP and reduced to integrated regions or “buckets” of equal width (0.01 ppm) corresponding to the region of δ 9.995 to δ −0.5. The regions between δ 4.865 and δ 4.775 were removed prior to statistical analyses thus eliminating any variability in suppression of the water signal. The signals corresponding 1 to d4 methanol (δ 3.335–δ 3.285) and d4 TSP (δ 0.00) were also removed at this stage. Data obtained from NMR and ESI-MS were imported into Microsoft Excel for the addition of labels. Data was imported into SIMCA-P 11 (Umetrics, Umea, Sweden) for multivariate analysis. All data were mean-centre scaled. PCA was carried out on all data sets. In order to determine differential metabolites accumulation data obtained from ESI-MS (postive mode) was imported into XLSTAT and subjected for one-way ANOVA statistical test and Benjamini-Hochberg Post hoc corrections procedure^62^.

### Metabolic pathways analysis

Metabolites showing signifcant accumulation compared to control treatment were used for pathway analysis. Data tables with metabolite peaks (mz/rt) were formatted as comma separated values (.csv) files and uploaded to the MetaboAnalyst 3.0 server (http://www.metaboanalyst.ca) for metabolic pathways analysis using Rice pathway libraries.

### Protein Profiling

Heat (12 h, 45 °C) (+/−5113), cold (6 h, −5 °C) (+/−5113), drought-stressed (7 d without water) (+/− 5113) and 5113-treated wheat were subjected to proteomic analysis through two-dimensional gel electrophoresis followed by MS analysis for several differentially expressed proteins. The proteomics analysis was conducted at the proteomics platform resources center, SciLifeLab, Uppsala University, Uppsala, Sweden.

Total proteins were extracted from two plant groups each representing a pool of 5 separate plants (200 mg frozen leaves tissue) using TCA-acetone protein precipitation^67^. The pellets were solubilized in 5 M urea, 2 M thiourea, 2% CHAPS, 2% SB3-10, and 20 mM dithiothreitol. Protein amount was quantified using 2-D Quant Kit (Amersham Biosciences, UK). For isoelectric focusing protein (250 µg) was loaded to a 24 cm Immobiline DryStrip Gel pH 3-10 NL (Amersham Biosciences). The strip was rehydrated with the sample for 12 h. Isoelectric focusing was carried out using Ettan IPGphor (Amersham Biosciences) with 500 V for 1 h, 1000 V for 1 h and 8000 V for 10:20 h. The strip was then equilibrated in: 6 M urea, 75 mM Tris-HCl pH 8.8, 29.3% glycerol, 2% SDS, 0.002% Bromophenol blue and 1% dithiothreitol for 15 min and for 15 min further after addition of 2.5% iodoacetamide.

SDS-PAGE used 12.5% acrylamide gels on an Ettan DALT Large Vertical electrophoresis system run at 1 W/gel overnight. Colloidal Coomassie Blue G-250 stained gels were scanned at 300 dpi resolution using a UMAX, PoweLook 1120 instrument (typical gel images representing protein profiles are provided in Fig. S4 and S5). The scanned images were uploaded into the image-analysis software Progenesis PG240 (Nonlinear Dynamics, USA) to annotate spots and calculate spot volumes for each gel where differences in abundance were displayed as normalized log ratios. The mass and isoelectric point (pI) was calculated for each spot using the same software.

Excised protein spots were washed with water, reduced by dithioerythritol and alkylated with iodoacetamide. Digestion used modified trypsin (Promega, USA) in 50 mM ammonium carbonate at 37 °C overnight. Peptides were extracted and analysed by MALDI-Tof/Tof analysis using a Bruker Ultraflex-Tof/Tof instrument. The peptide maps were used for searches in the NCBInr database using the Mascot search engine (MatrixScience.com) to identify the proteins. If needed for identification, MS/MS analysis was performed using the same instrument. MS/MS spectra were used to search against the NCBI non-redundant protein database using MS/MS Ion Search Engine, conducting protein identification based on matching the spectrum of a protein with a protein or DNA sequence database (http://www.matrixscience.com/search_form_select.html). The significance of the protein match with the ion score was based on the Mouse scoring algorithm.

### Statistical analysis

Data based on replicates were subjected to different statistics analysis methods using several packages. Analysis of variance (ANOVA) test to determine the significance between the different treatments was carried out using Costat (CoHort software, California, USA). SIMCA-P 11 (Umetrics, Umea, Sweden) was used for multivariate and PCA analysis for the metabolomics and proteomics analysis. The heat maps were generated based on using Pearson and Ward for distance measure and clustering algorithm using XLSTAT package^62^.

## Supplementary information

Supplementary Figures

Supplementary Table

## References

[1] Nakashima K, Ito Y, Yamaguchi-Shinozaki K (2009). Transcriptional regulatory networks in response to abiotic stresses in Arabidopsis and grasses. Plant Physiology.

[2] Zhu J-K (2016). Abiotic stress signaling and responses in plants. Cell.

[3] Zhang, R. *et al*. Evolution of disease defense genes and their regulators in plants. *International Journal of Molecular Science* **20**(2) (2019). 10.3390/ijms20020335 10.3390/ijms20020335 PMC6358896 30650550

[4] Huber AE, Bauerle TL (2016). Long-distance plant signaling pathways in response to multiple stressors: the gap in knowledge. Journal of Experimental Botany.

[5] Vishwakarma K (2017). Abscisic acid signaling and abiotic stress tolerance in plants: A review on current knowledge and future prospects. Frontiers in Plant Science.

[6] Takahashi F, Shinozaki K (2018). Long-distance signaling in plant stress response. Current Opinion in Plant Biology.

[7] Ramakrishna A, Ravishankar GA (2011). Influence of abiotic stress signals on secondary metabolites in plants. Plant Signaling & Behavior.

[8] Mundim FM, Pringle EG (2018). Whole-plant metabolic allocation under water stress. Frontiers in Plant Science.

[9] Yang D, Seaton DD, Krahmer J, Halliday KJ (2016). Photoreceptor effects on plant biomass, resource allocation, and metabolic state. Proceedings of the National Academy of Sciences USA.

[10] Hossain MA (2017). Heat or cold priming-induced cross-tolerance to abiotic stresses in plants: key regulators and possible mechanisms. Protoplasma.

[11] Lugtenberg B, Kamilova F (2009). Plant-growth-promoting rhizobacteria. Annual Review of Microbiology.

[12] Conrath U, Beckers GJ, Langenbach CJ, Jaskiewicz MR (2015). Priming for enhanced defense. Annual Review of Phytopathology.

[13] Dimkpa C, Weinand T, Asch F (2009). Plant–rhizobacteria interactions alleviate abiotic stress conditions. Plant, Cell &. Environment.

[14] Yang J, Kloepper JW, Ryu C-M (2009). Rhizosphere bacteria help plants tolerate abiotic stress. Trends in Plant Science.

[15] Ashraf M, Hasnain S, Berge O, Mahmood T (2004). Inoculating wheat seedlings with exopolysaccharide-producing bacteria restricts sodium uptake and stimulates plant growth under salt stress. Biology and Fertility of Soils.

[16] Creus CM, Sueldo RJ, Barassi CA (2004). Water relations and yield in Azospirillum-inoculated wheat exposed to drought in the field. Canadian Journal of Botany.

[17] Abd El-Daim IA, Bejai S, Meijer J (2014). Improved heat stress tolerance of wheat seedlings by bacterial seed treatment. Plant and Soil.

[18] Mishra P (2011). Alleviation of cold stress in inoculated wheat (Triticum aestivum L.) seedlings with psychrotolerant Pseudomonads from NW Himalayas. Archives of Microbiology.

[19] Pieterse CM (2014). Induced systemic resistance by beneficial microbes. Annual Review of Phytopathology.

[20] Egamberdieva D, Wirth SJ, Alqarawi AA, Abd Allah EF, Hashem A (2017). Phytohormones and beneficial microbes: essential components for plants to balance stress and fitness. Frontiers in Microbiology.

[21] Gamalero E, Glick BR (2015). Bacterial modulation of plant ethylene levels. Plant Physiology.

[22] Urano K, Kurihara Y, Seki M, Shinozaki K (2010). ‘Omics’ analyses of regulatory networks in plant abiotic stress responses. Current Opinion in Plant Biology.

[23] Wang Y, Ohara Y, Nakayashiki H, Tosa Y, Mayama S (2005). Microarray analysis of the gene expression profile induced by the endophytic plant growth-promoting rhizobacteria, Pseudomonas fluorescens FPT9601-T5 in Arabidopsis. Molecular Plant-Microbe Interactions.

[24] Mhlongo MI, Piater LA, Madala NE, Labuschagne N, Dubery IA (2018). The chemistry of plant-microbe interactions in the rhizosphere and the potential for metabolomics to reveal signaling related to defense priming and induced systemic resistance. Frontiers in Plant Science.

[25] Meena KK (2017). Abiotic stress responses and microbe-mediated mitigation in plants: the omics strategies. Frontiers in Plant Science.

[26] Khan N (2019). Comparative physiological and metabolic analysis reveals a complex mechanism involved in drought tolerance in chickpea (Cicer arietinum L.) induced by PGPR and PGRs. Scientific Reports.

[27] Kasim WA (2012). Control of drought stress in wheat using plant-growth-promoting bacteria. Journal of Plant Growth Regulation.

[28] Osman MEH (2014). Impact of bacterial priming on some stress tolerance mechanisms and growth of cold stressed wheat seedlings. International Journal of Plant Biology.

[29] Abd El-Daim IA, Bejai S, Fridborg I, Meijer J (2018). Identifying potential molecular factors involved in Bacillus amyloliquefaciens 5113 mediated abiotic stress tolerance in wheat. Plant Biology.

[30] Shelp BJ, Bown AW, Zarei A (2017). 4-Aminobutyrate (GABA): a metabolite and signal with practical significance. Botany.

[31] Ramesh SA (2015). GABA signalling modulates plant growth by directly regulating the activity of plant-specific anion transporters. Nature Communications.

[32] Carillo P (2018). GABA shunt in durum wheat. Frontiers in Plant Science.

[33] Vijayakumari K, Jisha KC, Puthur JT (2016). GABA/BABA priming: a means for enhancing abiotic stress tolerance potential of plants with less energy investments on defence cache. Acta Physiologiae Plantarum.

[34] Vijayakumari K, Puthur JT (2016). γ-Aminobutyric acid (GABA) priming enhances the osmotic stress tolerance in Piper nigrum Linn. plants subjected to PEG-induced stress. Plant Growth Regulation.

[35] Yong B (2017). Exogenous application of GABA improves PEG-induced drought tolerance positively associated with GABA-shunt, polyamines, and proline metabolism in white clover. Frontiers in Physiology.

[36] Jakab G (2005). Enhancing Arabidopsis salt and drought stress tolerance by chemical priming for its abscisic acid responses. Plant Physiology.

[37] Mierziak J, Kostyn K, Kulma A (2014). Flavonoids as important molecules of plant interactions with the environment. Molecules (Basel, Switzerland).

[38] Vacheron J (2013). Plant growth-promoting rhizobacteria and root system functioning. Frontiers in Plant Science.

[39] Chamam A (2013). Plant secondary metabolite profiling evidences strain-dependent effect in the Azospirillum–Oryza sativa association. Phytochemistry.

[40] Ashihara H, Stasolla C, Fujimura T, Crozier A (2018). Purine salvage in plants. Phytochemistry.

[41] Izaguirre-Mayoral ML, Lazarovits G, Bara B (2018). Ureide metabolism in plant-associated bacteria: purine plant-bacteria interactive scenarios under nitrogen deficiency. Plant and Soil.

[42] Batista-Silva W (2019). The role of amino acid metabolism during abiotic stress release. Plant, Cell &. Environment.

[43] Hayat S (2012). Role of proline under changing environments: a review. Plant Signaling & Behavior.

[44] Du N (2016). Proteomic analysis reveals the positive roles of the plant-growth-promoting rhizobacterium NSY50 in the response of cucumber roots to Fusarium oxysporum f. sp. cucumerinum inoculation. Frontiers in Plant Science.

[45] Kwon YS (2016). Proteomic analyses of the interaction between the plant-growth promoting rhizobacterium Paenibacillus polymyxa E681 and Arabidopsis thaliana. Proteomics.

[46] Singh RP, Runthala A, Khan S, Jha PN (2017). Quantitative proteomics analysis reveals the tolerance of wheat to salt stress in response to Enterobacter cloacae SBP-8. PloS One.

[47] Niazi A (2014). Genome analysis of Bacillus amyloliquefaciens subsp. plantarum UCMB5113: A rhizobacterium that improves plant growth and stress management. PloS One.

[48] Johansson AH (2014). Studies of plant colonisation by closely related Bacillus amyloliquefaciens biocontrol agents using strain specific quantitative PCR assays. Antonie van Leeuwenhoek.

[49] Yamori W (2016). Photosynthetic response to fluctuating environments and photoprotective strategies under abiotic stress. Journal of Plant Research.

[50] Kandasamy S (2009). Understanding the molecular basis of plant growth promotional effect of Pseudomonas fluorescens on rice through protein profiling. Proteome Science.

[51] Timperio AM, Egidi MG, Zolla L (2008). Proteomics applied on plant abiotic stresses: Role of heat shock proteins (HSP). Journal of Proteomics.

[52] Wang W, Vinocur B, Shoseyov O, Altman A (2004). Role of plant heat-shock proteins and molecular chaperones in the abiotic stress response. Trends in Plant Science.

[53] Vinocur B, Altman A (2005). Recent advances in engineering plant tolerance to abiotic stress: achievements and limitations. Current Opinion in Biotechnology.

[54] Komatsu S, Kamal AHM, Hossain Z (2014). Wheat proteomics: proteome modulation and abiotic stress acclimation. Frontiers in Plant Science.

[55] Alptekin B, Langridge P, Budak H (2017). Abiotic stress miRNomes in the Triticeae. Functional and Integrative Genomics.

[56] Cho S-M, Kang BR, Kim YC (2013). Transcriptome analysis of induced systemic drought tolerance elicited by Pseudomonas chlororaphis O6 in Arabidopsis thaliana. The Plant Pathology Journal.

[57] Timmusk S (2014). Drought-tolerance of wheat improved by rhizosphere bacteria from harsh environments: enhanced biomass production and reduced emissions of stress volatiles. PLoS One.

[58] Barkla BJ (2016). Identification of abiotic stress protein biomarkers by proteomic screening of crop cultivar diversity. Proteomes.

[59] Backer R (2018). Plant growth-promoting rhizobacteria: context, mechanisms of action, and roadmap to commercialization of biostimulants for sustainable agriculture. Frontiers in Plant Science.

[60] Miché L, Balandreau J (2001). Effects of rice seed surface sterilization with hypochlorite on inoculated Burkholderia vietnamiensis. Applied and Environmental Microbiology.

[61] Dunlap CA, Kim S-J, Kwon S-W, Rooney AP (2016). Bacillus velezensis is not a later heterotypic synonym of Bacillus amyloliquefaciens; Bacillus methylotrophicus, Bacillus amyloliquefaciens subsp. plantarum and ‘Bacillus oryzicola’ are later heterotypic synonyms of Bacillus velezensis based on phylogenomics. International Journal of Systematic and Evolutionary Microbiology.

[62] Addinsoft. XLSTAT statistical and data analysis solution, https://www.xlstat.com. (2019).

[63] Shah SH, Houborg R, McCabe MF (2017). Response of chlorophyll, carotenoid and SPAD-502 measurement to salinity and nutrient stress in wheat (Triticum aestivum L.). Agronomy.

[64] Murshed R, Lopez-Lauri F, Sallanon H (2008). Microplate quantification of enzymes of the plant ascorbate-glutathione cycle. Analytical Biochemistry.

[65] Ward JL (2010). An inter-laboratory comparison demonstrates that [1H]-NMR metabolite fingerprinting is a robust technique for collaborative plant metabolomic data collection. Metabolomics.

[66] Ward JL, Harris C, Lewis J, Beale MH (2003). Assessment of 1H NMR spectroscopy and multivariate analysis as a technique for metabolite fingerprinting of Arabidopsis thaliana. Phytochemistry.

[67] Méchin, V., Damerval, C. & Zivy, M. In Plant Proteomics Vol. 355 *Methods in Molecular Biology* (eds Hervé. Thiellement, Michel. Zivy, Catherine. Damerval, & Valérie. Méchin) Ch. 1, 1–8 (Humana Press, 2007). 10.1385/1-59745-227-0:1 17093296

