# Supplementary material for: Bacillus velezensis 5113 Induced Metabolic and Molecular Reprogramming during Abiotic Stress Tolerance in Wheat
Source: Sci Rep. 2019 Nov 8;9:16282. doi: 10.1038/s41598-019-52567-x (PMC6841942; doi:10.1038/s41598-019-52567-x)
Supplement: Supplementary file 1 — Supplementary Figures [file 41598_2019_52567_MOESM1_ESM.pdf]

Bacillus velezensis 5113 Induced Metabolic and Molecular Reprogramming during Abiotic Stress Tolerance in Wheat.

Islam A. Abd El-Daim, Sarosh Bejai and Johan Meijer

Supplementary Figures

Fig. S1: Effect of abiotic stress factors (heat, cold and drought) on the plant ascorbate-glutathione redox cycle in the leaves of 5113-treated wheat seedlings.

Fig. S2: Principle component analysis of metabolome effects of 5113 treatment and drought stress as 7 days without water.

Fig. S3: Principle component analysis of metabolome effects of 5113 treatment and cold stress as 12h at -5 ^{o}C.

Fig. S4: Typical two-dimensional gel profiles of proteins from wheat leaves after bacterial inoculation with 5113 and abiotic stress treatments (heat and cold).

Fig. S5: Typical two-dimensional gel profiles of proteins from wheat leaves after bacterial inoculation with 5113 and abiotic stress treatment (drought stress).

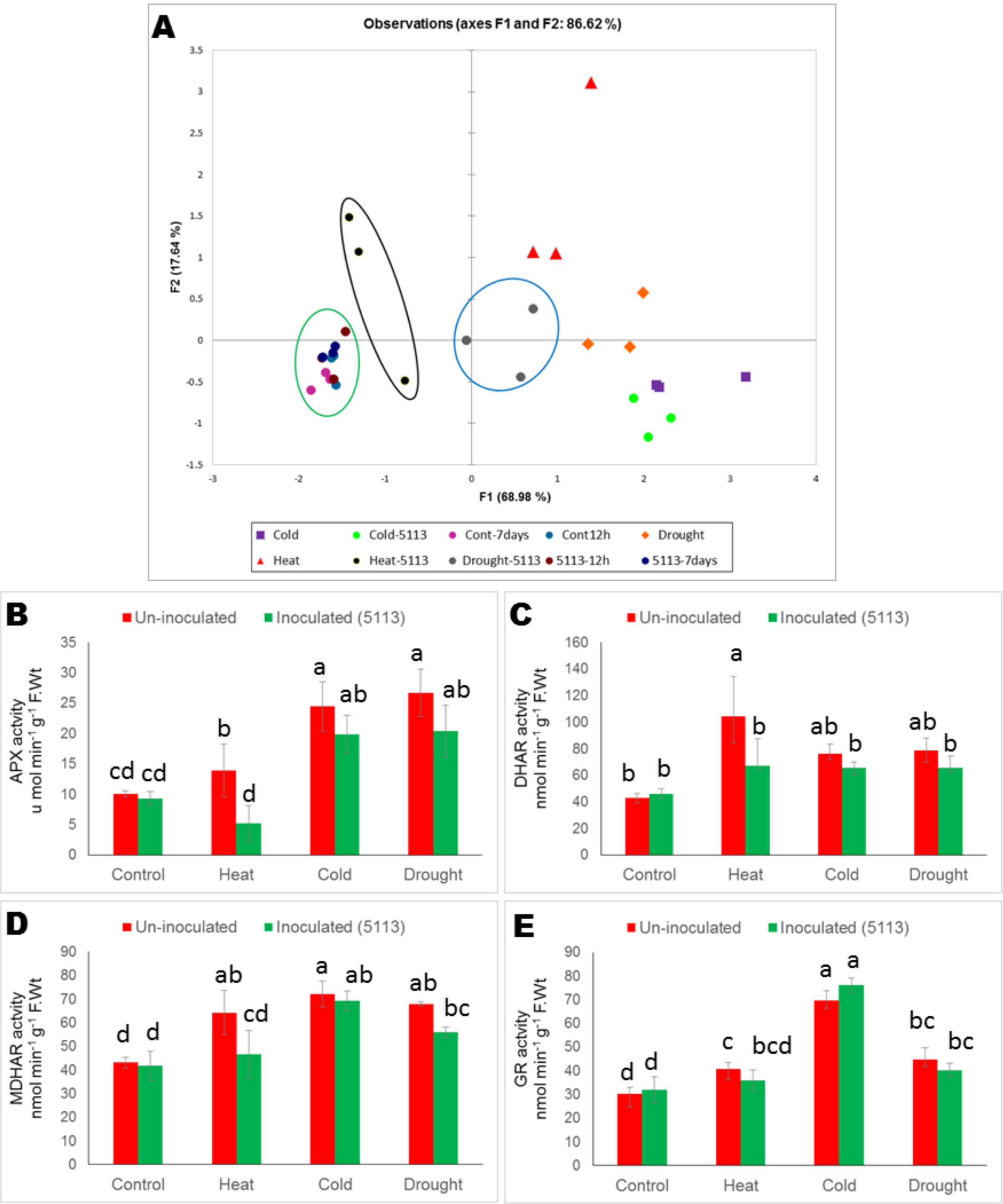

Fig. S1: Effect of abiotic stress factors (heat, cold and drought) on the plant ascorbate-glutathione redox cycle in the leaves of 5113-treated wheat seedlings. **A.** PCA analysis for the activities of the enzymes APX **(B)**, DHAR **(C)**, MDHAR **(D)** and GR **(E)**. Bars indicate standard deviation between 3 replicates. Treatments labelled with identical letters are not significant at $p<0.05$

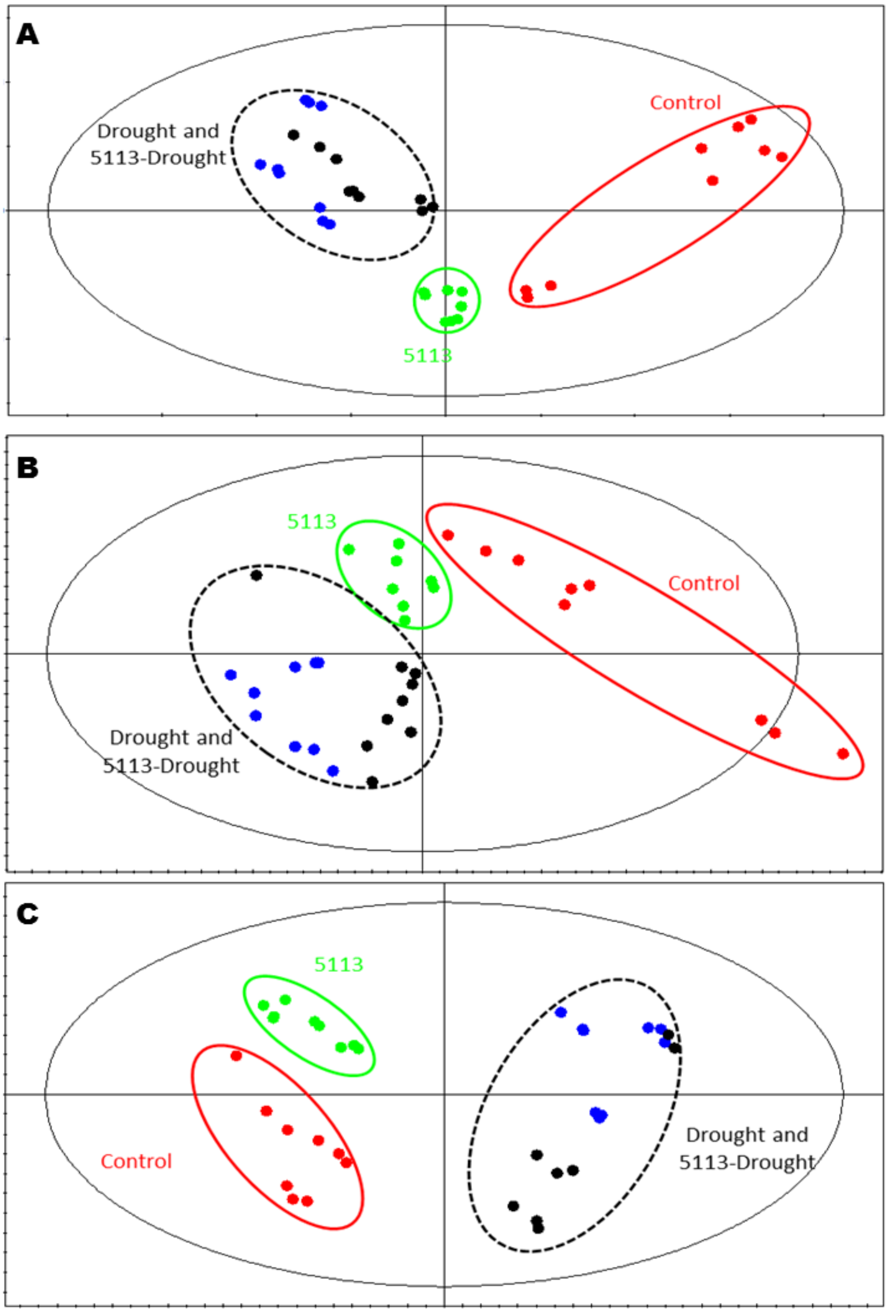

Fig. S2: Principle Component Analysis (PCA) of metabolome effects of 5113 treatment and 7 days without water (drought stress). Plant samples represented untreated plants (red dots); 5113-treated (green dots), drought stress (blue dots); or drought-stressed and 5113-treated leaves (black dots). Analysis was based on positive mode ESI-MS **(A)**; negative mode ESI-MS **(B)** and NMR **(C)**. All treatments in these analyses were represented by 9 data points (three biological samples and three technical repeats)

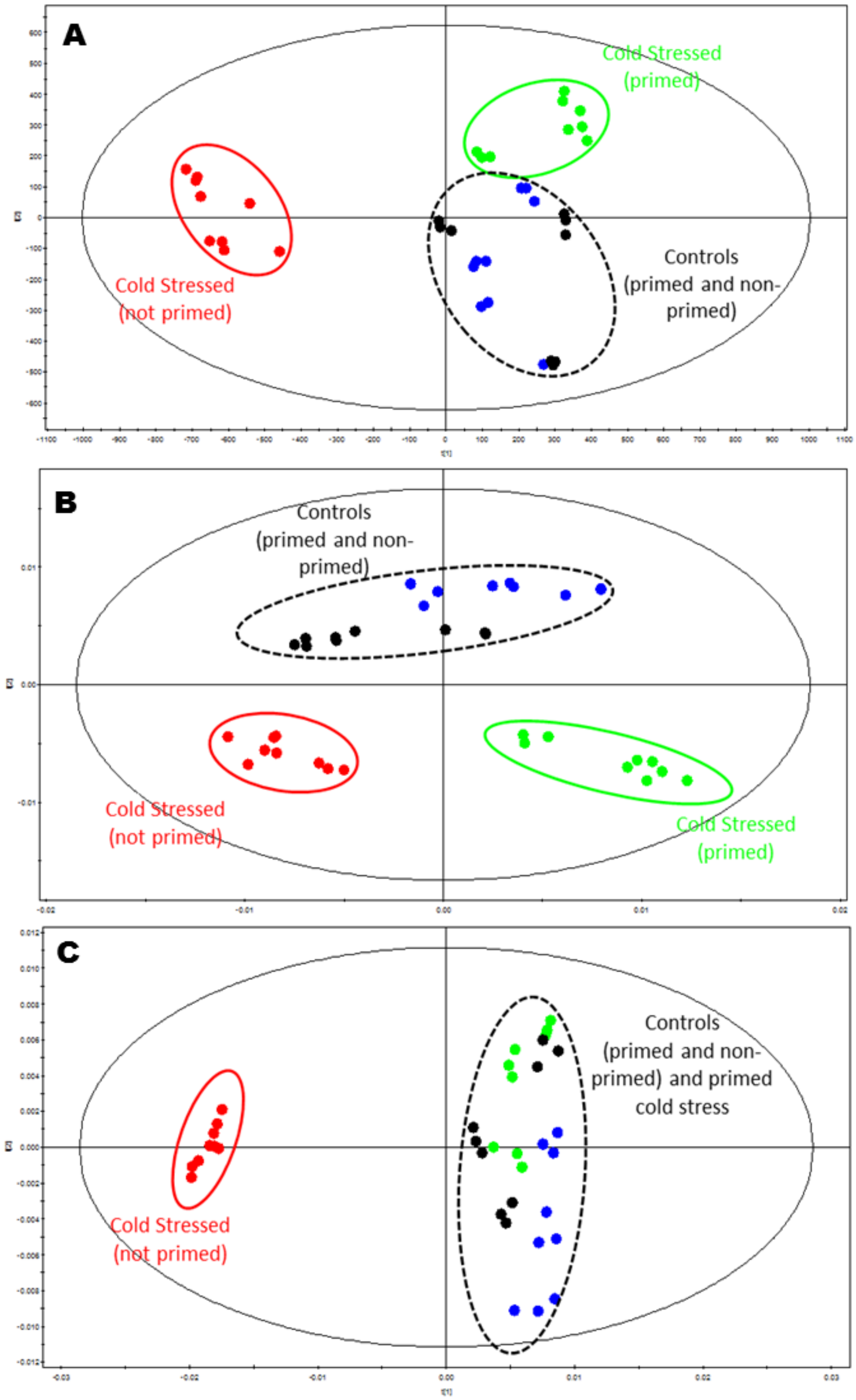

Fig. S3: Principle Component Analysis (PCA) of metabolome effects of 5113 treatment and cold stress as 12 h at -5 ºC. Plant samples represented untreated plants (red dots); 5113-treated (green dots), cold stress (blue dots); or cold-stressed and 5113-treated leaves (black dots). Analysis was based on positive mode ESI-MS **(A)**; negative mode ESI-MS **(B)** and NMR **(C)**. All treatments in these analyses were represented by 9 data points (three biological samples and three technical repeats)

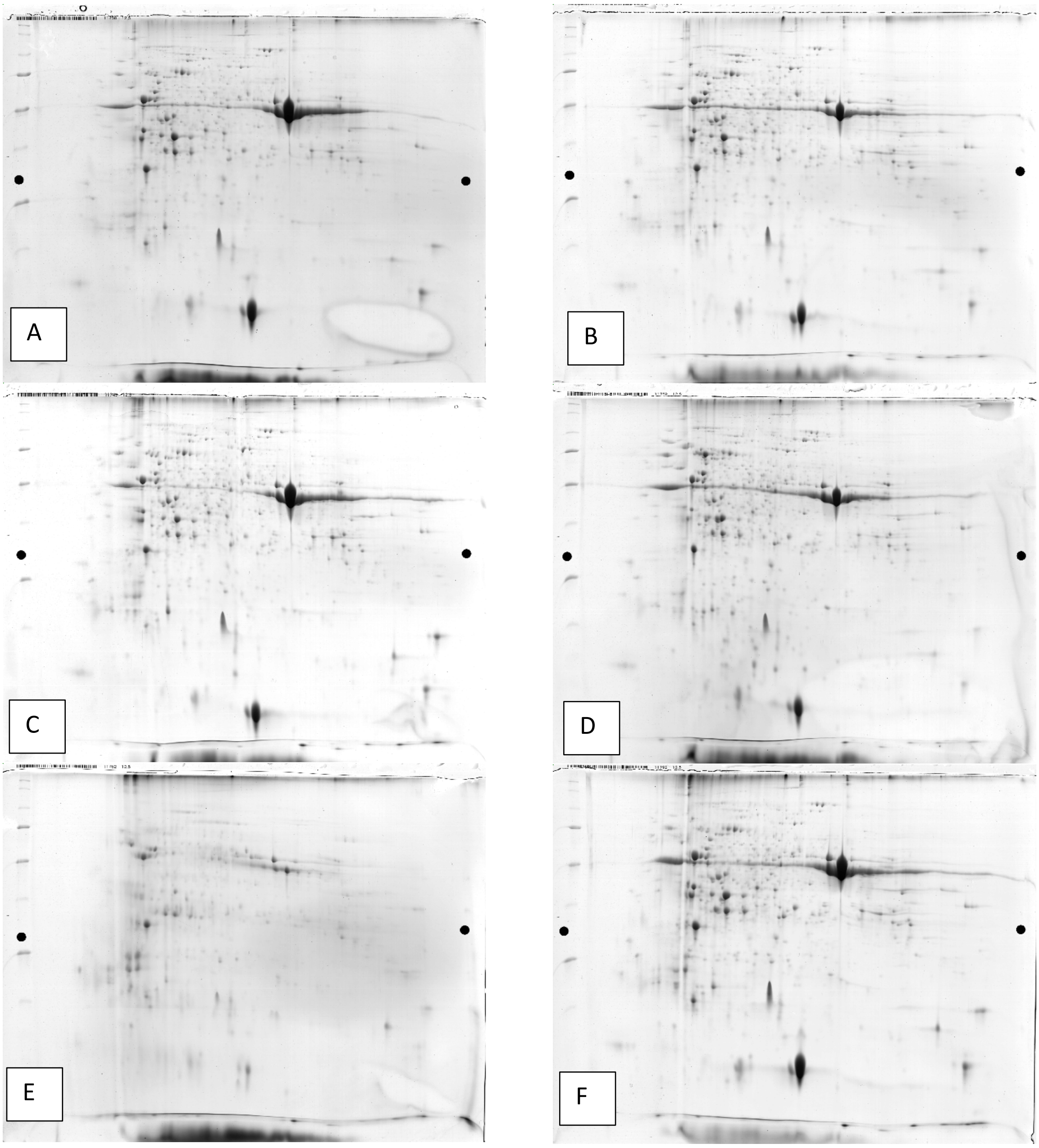

Fig. S4: Typical (un-processed) two-dimensional gel profiles of protein from wheat leaves after bacterial inoculation with 5113 and abiotic stress treatments (heat and cold). **A:** Control, **B:** Control inoculated, **C:** Heat-stressed (12h at 45°C), **D:** Heat-stressed inoculated (12h at 45°C), **E:** Cold-stressed (12h at -5°C), **F:** Cold-stressed inoculated (12h at -5°C).

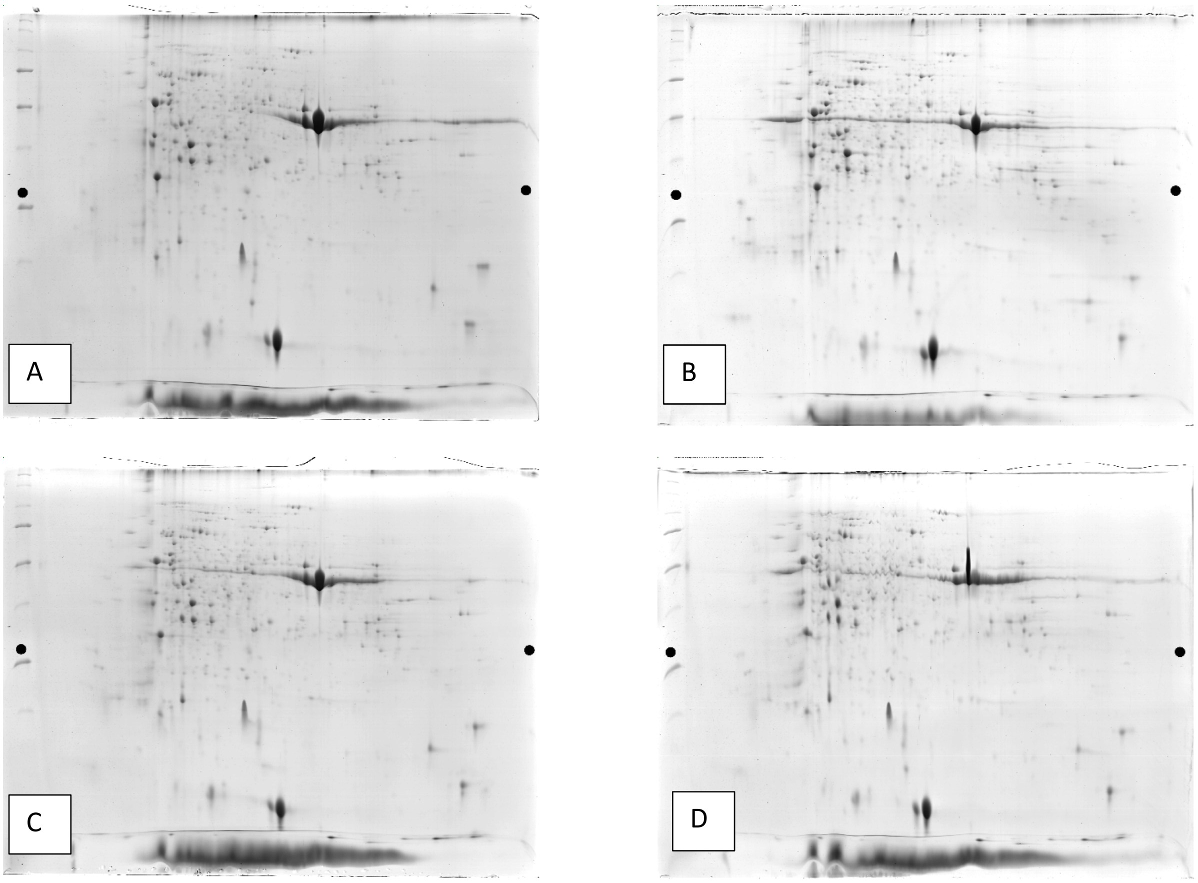

Fig. S5: Typical (un-processed) two-dimensional gel profiles of proteins from wheat leaves after bacterial inoculation with 5113 and drought stress **A:** Control, **B:** Control inoculated, **C:** Drought-stressed (7 days without water) and **D:** Drought-stressed inoculated (7 days without water).
