# Supplementary material for: Bacillus velezensis 5113 Induced Metabolic and Molecular Reprogramming during Abiotic Stress Tolerance in Wheat
Source: Sci Rep. 2019 Nov 8;9:16282. doi: 10.1038/s41598-019-52567-x (PMC6841942; doi:10.1038/s41598-019-52567-x)
Supplement: Supplementary file 2 — Supplementary Table [file 41598_2019_52567_MOESM2_ESM.pdf]

Bacillus velezensis 5113 Induced Metabolic and Molecular Reprogramming during Abiotic Stress Tolerance in Wheat.

Islam A. Abd El-Daim, Sarosh Bejai and Johan Meijer

Supplementary Table

Table S1: Further data from protein analysis. Differentially expressed proteins were analyzed by peptide mapping and/or sequence analysis using mass spectrometry.

ID	MASCOT value	% coverage[a]	mass values[b] searched	mass values[b] matched	Peptide sequence[c]
3	142	18	30	17	
55	194	38	59	15	
137	204	37	42	20	
150	30	2			SNCFHASDDK
155	158	56	42	14	
166	70	16	23	7	
176	93	23	32	9	
192	93	41	54	9	
194	75	21	23	9	
202	85	36	21	6	
203	128	39	40	12	
206	36	3			YQAFELIHAR
218	45	3			IAYQFYR
238	145	46	32	16	
242	42	3			FKESEIYHCR
269	103	50	19	7	
271	47	1			QDGSPSVGEK
285	86	33	20	6	

[a] Indicates the protein sequence coverage by the matching peptide fragments or peptide sequences.

[b] Indicates the number of mass values searched with and hits (including methionine modifications, while carbaimidomethyl groups were fixed)and with peptide mass tolerance +/- 0.05 Da.

[c] Amino acid sequences derived by MS-MS analysis of peptides.
